# A new perspective on membrane-embedded Bax oligomers using DEER and bioresistant orthogonal spin labels

**DOI:** 10.1038/s41598-019-49370-z

**Published:** 2019-09-10

**Authors:** Markus Teucher, Hui Zhang, Verian Bader, Konstanze F. Winklhofer, Ana J. García-Sáez, Andrzej Rajca, Stephanie Bleicken, Enrica Bordignon

**Affiliations:** 1 Faculty of Chemistry and Biochemistry, Ruhr University Bochum, Bochum, Germany; 2 Department of Chemistry, University of Nebraska, Lincoln, Nebraska USA; 3 Institute of Biochemistry and Pathobiochemistry, Ruhr University Bochum, Bochum, Germany; 4 Interfaculty Institute of Biochemistry, Eberhard Karls University Tübingen, Tübingen, Germany; 5 ZEMOS, Ruhr University Bochum, Bochum, Germany

**Keywords:** Molecular conformation, NMR spectroscopy

## Abstract

Bax is a Bcl-2 protein crucial for apoptosis initiation and execution, whose active conformation is only partially understood. Dipolar EPR spectroscopy has proven to be a valuable tool to determine coarse-grained models of membrane-embedded Bcl-2 proteins. Here we show how the combination of spectroscopically distinguishable nitroxide and gadolinium spin labels and Double Electron-Electron Resonance can help to gain new insights into the quaternary structure of active, membrane-embedded Bax oligomers. We show that attaching labels bulkier than the conventional MTSL may affect Bax fold and activity, depending on the protein/label combination. However, we identified a suitable pair of spectroscopically distinguishable labels, which allows to study complex distance networks in the oligomers that could not be disentangled before. Additionally, we compared the stability of the different spin-labeled protein variants in *E*. *coli* and HeLa cell extracts. We found that the *gem*-diethyl nitroxide-labeled Bax variants were reasonably stable in HeLa cell extracts. However, when transferred into human cells, Bax was found to be mislocalized, thus preventing its characterization in a physiological environment. The successful use of spectroscopically distinguishable labels on membrane-embedded Bax-oligomers opens an exciting new path towards structure determination of membrane-embedded homo- or hetero-oligomeric Bcl-2 proteins via EPR.

## Introduction

Apoptosis is a form of programmed cell death pivotal for mammals. Aberrant apoptosis is involved in severe diseases like cancer, stroke, myocardial infarction or autoimmune, as well as neurodegenerative diseases^1–3^. The interaction of the pro- and anti-apoptotic Bcl-2 protein partners plays a crucial role in apoptosis regulation and execution^4–6^. Therefore, these proteins are interesting targets for drug development^4,7–10^ and the first drug specifically targeting a Bcl-2 protein (Venetoclax) was recently approved by the United States Food and Drug Administration for treatment of chronic lymphocytic leukemia. The development of Venetoclax was strongly aided by X-ray structures of anti-apoptotic Bcl-2 proteins with activating BH3-only peptides and later Venetoclax or its precursors^8,11^, which proves the importance of structural models of proteins for drug design.

**Figure 1:**
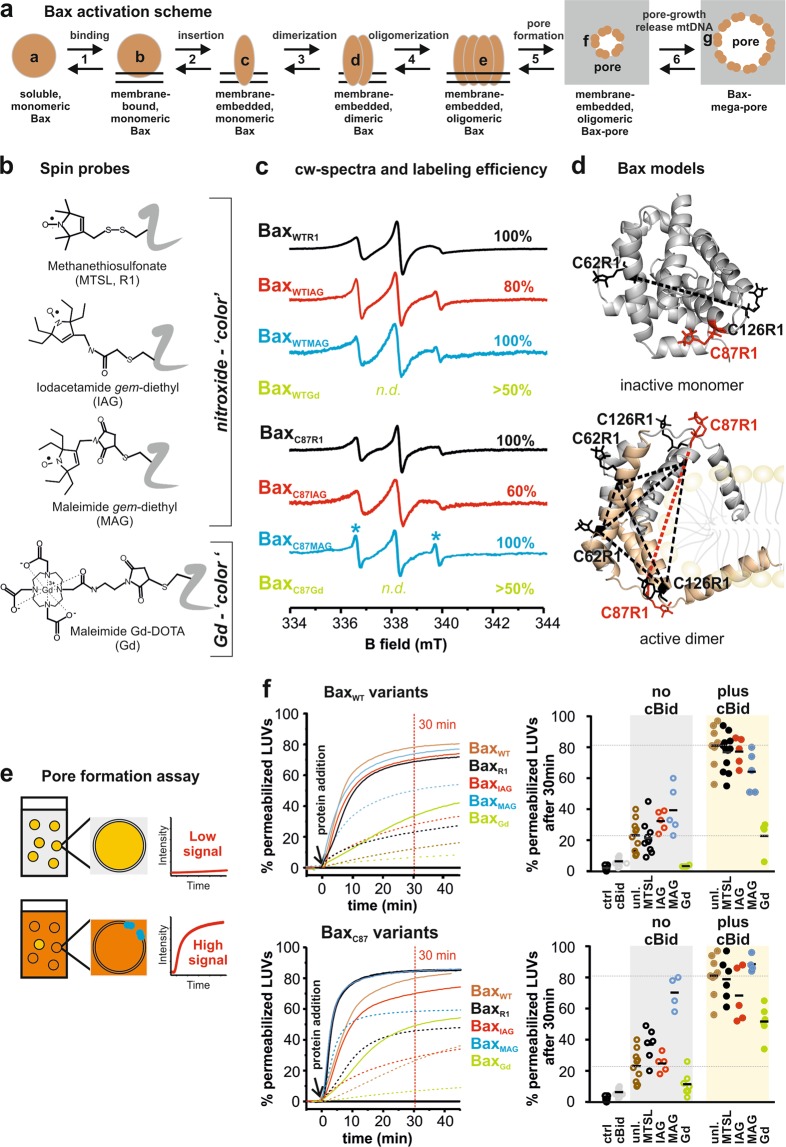
Bax activation scheme, spin labeling and activity assays. (**a**) Sketch of conformational changes of Bax during activation. The protein exists at least in seven conformational states (*a-g*). Soluble monomeric Bax (state “*a*”) can transiently interact with the mitochondrial outer membrane MOM (“*b*”). Upon interaction with an activator-type BH3-only protein membrane insertion is induced (“*c*”)^20,22^ followed by the formation of homo-dimers (“*d*”) that assemble into higher order oligomers^10,18,22,24^ (“*e*”). The oligomers form a toroidal pore in the MOM allowing the release of Cyt c^39,59^ (“*f*”). These pores can grow to “mega-pores” able to release mitochondrial DNA^14,17^. (**b**) The four spin labels used in this study, targeting cysteine residues. (**c**) Room temperature cw spectra of the nitroxide-labeled proteins, with the degree of labeling (spin/cysteine ratio) indicated in %. Asterisks indicate residual free label. For Gd-labeled Bax see Supp. Fig. 2. (**d**) Structural models of monomeric^12^ and dimeric active Bax^18^ with one MTSL (R1) label attached per site using the software MMM^55^. Intra- and inter- monomer distances are highlighted by dotted lines (black for the wild type variants, red for the C87 variants). (**e**) Graphical scheme of the assay used to address Bax-induced pore formation. (**f**) Kinetic curves of pore formation (left panels) and data points after 30 min (right panels) for the Bax_WT_ variants (upper panels) and for the Bax_C87_ variants (lower panels), both compared to unlabeled Bax_WT_. Dotted lines: Bax alone (50 nM) with vesicles; solid lines: 50 nM Bax with 50 nM cBid and vesicles. Note that the maximally reachable fraction of permeabilized vesicles varies for different experiments, depending on the lipid preparation (100% permeabilization was detected by adding detergent).

Here we focus on the pro-apoptotic Bcl-2 protein Bax, which exists in two major conformations. In healthy cells it is mainly a soluble, monomeric, inactive protein whose structure is known^12^, while upon a pro-apoptotic stimulus Bax transforms into the active pore-forming, membrane-embedded oligomer, whose structure is only partially known (recently reviewed in^13^). The pores formed by active Bax enable mitochondrial outer membrane permeabilization and apoptosis execution^4,13^ and the formation of “mega-pores” (Fig. 1a) is likely related to the generation of pro-inflammatory signals^14–17^. Therefore, Bax is a central hub for the health of cells, tissues and organs.

Structure determination of active Bax has proven to be difficult due to its membrane-embedded nature, its engagement in homo- and hetero-oligomers^18–22^, its inhomogeneous oligomer size^23–25^, the complicated multistep transition during activation^22,24,26–29^ (Fig. 1a) and the formation of “off-pathway” swapped-dimers in *in vitro* conditions^18,20^. In our previous work we established an experimental setup to study Bax structure by covalently attaching one or two nitroxide spin probes ((1-Oxyl-2,2,5,5-tetramethyl-∆3-pyrroline-3-methyl) methanethiosulfonate, MTSL) and retrieving intra- and inter-monomer distances on more than 40 protein variants^18^ using Double Electron-Electron Resonance (DEER). The study of spin-labeled Bax homo-oligomers by DEER faces many challenges. First, Bax oligomerization creates complex protein systems with multiple intra- and inter- dimer spin distances that are difficult to assign when analyzing singly-labeled proteins. Notably, cross-link studies face the same problem (for a recent review see^13^). To simplify the spin system and obtain distance constraints within each monomer in the oligomer, we used doubly-labeled proteins spin-diluted with unlabeled wild type partners^18^, which enhanced the intra-monomer distance within the distance distribution. Based on these data we could propose a coarse-grained model of active Bax^13^. Additionally, the conventional nitroxide labels were found to be incompatible with cellular extracts or isolated mitochondria, as intra-cellular agents can chemically reduce the nitroxide group to the EPR silent hydroxylamine or can release the labels from the protein after reduction of the S-S linker^18,30^.

To increase the information content that can be obtained from DEER experiments, we explore the use of spectroscopically distinguishable nitroxide and gadolinium spin probes (called two ‘colors’ for simplicity, see Fig. 1b and Supp. Fig. 1) on membrane-embedded, oligomeric Bax. We used a ‘two-color-three-channel’ DEER strategy, which is, in a nutshell, similar to a FRET (Förster resonance energy transfer) experiment. DEER is performed with two distinguishable spin labels excited with different microwave frequencies, while FRET operates via two fluorophores absorbing and emitting at different frequencies. However, in contrast to FRET, DEER can probe not only the mean distance between “donor and acceptor” pairs but yields precise distributions of distances between “donor-acceptor”, “acceptor-acceptor” and “donor-donor” pairs (reviewed in^31^). Both techniques rely on the dipole-dipole coupling between the two fluorophores or the two spins which depends on the distance r between them. FRET can be performed at room temperature and has a r^−6^ dependence, DEER at cryogenic temperature and has a r^−3^ dependence. A schematic description of DEER with two ‘colors’ is shown in Supp. Fig. 1.

To address if it is possible to use a ‘two-color’ DEER strategy also in a cellular environment, we would need to label Bax with labels that are bio- as well as Bax-compatible. To this end, we labeled a single- and a double cysteine variant of Bax with a maleimide Gd-DOTA label (known to be biocompatible^32,33^) as well as with three nitroxide spin probes: the conventional MTSL and the sterically-shielded iodacetamide- and maleimide- *gem*-diethyl nitroxide IAG and MAG^34^ (suggested to be more resistant towards reducing agents than MTSL^35–37^) (Fig. 1b–d). We addressed the compatibility of the different labels with Bax fold and activity, and the stability of the spin-labeled Bax variants in a cellular context using *E*. *coli* or HeLa cell extracts. Once inserted into HeLa cells, Bax did not localize in the correct subcellular compartment, preventing its DEER characterization under physiological conditions. However, by using spectroscopically distinguishable labels combined with DEER, we were able to assign distances arising from a complex spin system that could not be assigned by using only one type of label. In summary, we could show the first proof-of-principle DEER study of active oligomeric Bax proteins labeled with HeLa-biocompatible spectroscopically orthogonal spin labels.

## Results

### Spin labeling and activity of Bax variants

In this study we used Bax wild type (Bax_WT_), which has two native cysteines at positions 62 and 126 and the mutant Bax_C87(C62S, C126S)_ which has a single surface-exposed cysteine (Fig. 1d). We chose these two MTSL-labeled variants because we previously showed that they are active and well folded in both inactive and active conformations and their interspin distance distributions are clearly distinguishable in both conformations^18,23^.

The two protein variants were labeled with MTSL, IAG, MAG and maleimide-Gd-DOTA labels and room temperature continuous wave (cw) X-band spectra of the three nitroxide-labeled proteins revealed similar spectral features and a high degree of labeling (Fig. 1c). Due to the large spectral width, X-band cw spectra cannot be detected for the Gd-DOTA labeled proteins at the used concentrations, thus we estimated the labeling efficiency by echo-detected Q-band field swept spectra at cryogenic temperatures (Supp. Fig. 2).

Point mutations as well as label attachment can affect Bax fold and function^13,18^. Therefore, we analyzed the pore-forming activity of all spin-labeled Bax variants by kinetic experiments following fluorophore release from liposomes (Fig. 1e,f). The membrane impermeable fluorophore “calcein” was entrapped in unilamellar liposomes at high, self-quenching concentration. Upon Bax-induced pore formation, calcein is released from the liposomes and the fluorescence intensity increases (Fig. 1e). The data were normalized to minimal (liposomes in absence of protein and detergent) and maximal (upon detergent addition to destroy all liposomes) fluorescence values. The fluorophore release curves of all Bax variants were compared to the unlabeled wild type protein, which revealed that spin labeling may affect Bax activity. Figure 1f shows a direct comparison of kinetic experiments performed in parallel (left panel) on the same lipid batch, as well as a comparison of independent experimental repetitions (right panel). Both data representations are important as the first will better reveal kinetic details, and the second the variability in terms of auto-activity and maximal permeabilization with different protein and liposome batches.

For Bax_WT_, we observed almost no effects in pore forming activity upon labeling with MTSL; IAG labeling induced a slight increase in auto-activity. MAG labeling induced bigger effects: it produced a variant that was partially auto active and not fully responsive to cBid addition. Finally, labeling with Gd-DOTA strongly reduced Bax_WT_ activity. Labeling of Bax_C87_ with MTSL and IAG mildly increased auto-activity, while MAG labeling produced a strongly auto-active Bax variant. Overall, Bax_C87_ seems to be slightly more active than Bax_WT_. Attaching maleimide Gd-DOTA to position 87 clearly reduced auto-activity as well as maximal activity upon cBid addition, however Bax_C87Gd_ was still reasonable active.

In summary, spin labeling can clearly affect Bax activity, but except for Bax_WTGd,_ Bax_WTMAG_ and Bax_C87MAG_, all protein variants showed reasonable activity and an acceptably low auto-activity. A summary of the results obtained for the different protein variants is given in Supp. Table 1. Interestingly, labeling with the nitroxide probe (especially MAG) often enhanced the auto-activity of Bax, while maleimide Gd-DOTA had the opposite effect.

### From the inactive to the active conformation

The detected alterations in the pore forming activity could be caused by label-induced structural changes preventing Bax from adopting its inactive or active conformations^13^. To validate the fold of all spin-labeled protein variants in both states, the intra- and inter-monomeric distances were measured by DEER. The results were compared to distance simulations performed on the available structural models shown in Fig. 1d^12,18,20^.

**Figure 2:**
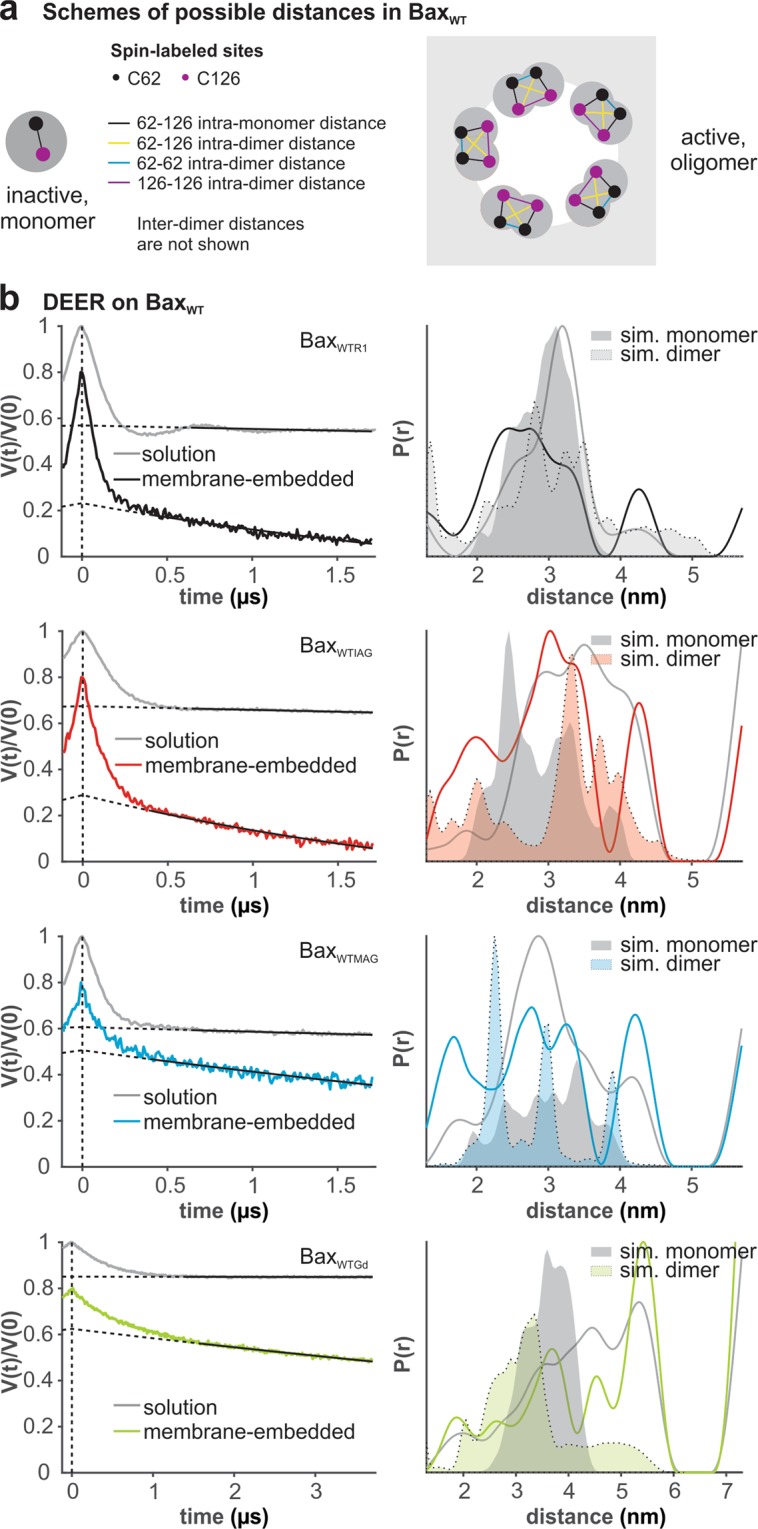
DEER data of spin-labeled Bax_WT_ variants in solution and in membranes. (**a**) Schemes of possible interspin distances. (**b**) Primary data with background functions (left) and distance distributions (right) in the monomeric (solution) and oligomeric (membrane-embedded) conformations obtained by Tikhonov regularization with DeerAnalysis2018^54^. Shaded areas present simulations of intra-monomer and intra-dimer distances based on the structures in^12,18,20^. Data evaluation and validation is provided in Supp. Figs 4–6.

Figure 2 shows the DEER analysis of inactive and active Bax_WT_ variants (see also Supp. Fig. 3). In line with the simulations and our earlier work^18,23^, we found a mean interspin distance between positions 62 and 126 of about 3 nm in monomeric Bax_WTR1_ (Fig. 2b, grey). The variants Bax_WTIAG_ and Bax_WTMAG_ showed mean distances peaking at 3.5 and 3 nm, with the IAG and MAG variants having a larger distribution width (Fig. 2b, grey). For the nitroxide labels, the average distance distributions simulated on the 20 NMR models of monomeric Bax were found to be compatible with the experimental distances, indicating that all three variants are properly folded. However, we found a large variation in inter-spin distances among the different models, due to the varying steric constraints experienced by the label rotamers (Supp. Fig. 4).

Notably, we decided to keep the length of all nitroxide DEER traces at 1.7 µs to have the possibility to consistently compare the primary data with the same dipolar evolution time in all conditions (aqueous and membrane environments). The short trace length was dictated by the low protein concentrations and the fast spin relaxation times in membranes, thereby the reliability of the distance analysis is limited to 5 nm^38^. We could not further concentrate the MAG-labeled protein versions as they were prone to aggregation. Therefore, we decided to use protein concentrations of about 20 µM, which minimizes protein aggregation as well as the impact of the background decay function on the reliability of the extracted distances, as shown by comparison of long and short DEER traces in Supp. Fig. 3. A validation of the distance distributions is shown in Supp. Fig. 3.

Bax_WTGd_ showed a very broad distance distribution from 2 to 6 nm, which is incompatible with the globular fold of Bax and the corresponding distance simulations (Fig. 2 and Supp. Fig. 4). Therefore, we concluded that the maleimide Gd-DOTA label destabilizes the protein, which is correlated with its low activity (Fig. 1f). The residual observed activity is likely due to an existing fraction of the unlabeled protein.

Next, we addressed if the transformation into the oligomeric form can be affected by the different labels. Membrane insertion and oligomerization of all Bax variants was induced by incubation with liposomes and cBid. The protein-containing liposomes were then separated from residual non-inserted Bax by centrifugation. Based on the EPR spectra detected on the pellet fractions (Supp. Fig. 5), the majority of Bax was found in the pellet, except for Bax_WTGd_.

When Bax_WT_ variants oligomerize at the membrane, there are two spins per monomeric unit and thus multiple intra and inter-monomer distances appear, as schematically shown in Fig. 2a. To further complicate the assignment of the multiple distances, a structural model exists only for the dimeric unit^18,20^. The DEER time traces detected in the pellet fraction for all variants are shown in Fig. 2b. Despite the steeper decay of the background function in membrane environments, the validation of the distance distributions provided a good level of confidence in the distances <5 nm (see validation in Supp. Fig. 6).

As visible from the primary DEER data and in agreement with previous studies, active membrane-embedded Bax_WTR1_ shows a broader distance distribution than the monomeric form (Fig. 2b), which is consistent with the structural model^13,18,20^. Similar changes in the overall distance distribution are also observed for Bax_WTIAG_ and Bax_WTMAG_ upon membrane insertion. The simulations performed on the dimer would predict the experimentally observed increase in the short distances (due mainly to the 62–62 interaction between the BH3 domains). However, the multi-spin distance distributions are too broad and complex to reliably assess the protein fold in the doubly-labeled variants at the membrane.

For the Gd variants, traces of 3.7 µs were recorded, yielding a large distance distribution similar to what was found in aqueous environment, which gave strength to the suggestion that Bax_WTGd_ cannot switch to its active conformation at the membrane. Based on DEER analysis and activity assays, we concluded that Bax_WTR1_ and Bax_WTIAG_ adopt the correct oligomeric configuration at the membrane and are suitable labels for Bax_WT_.

**Figure 3:**
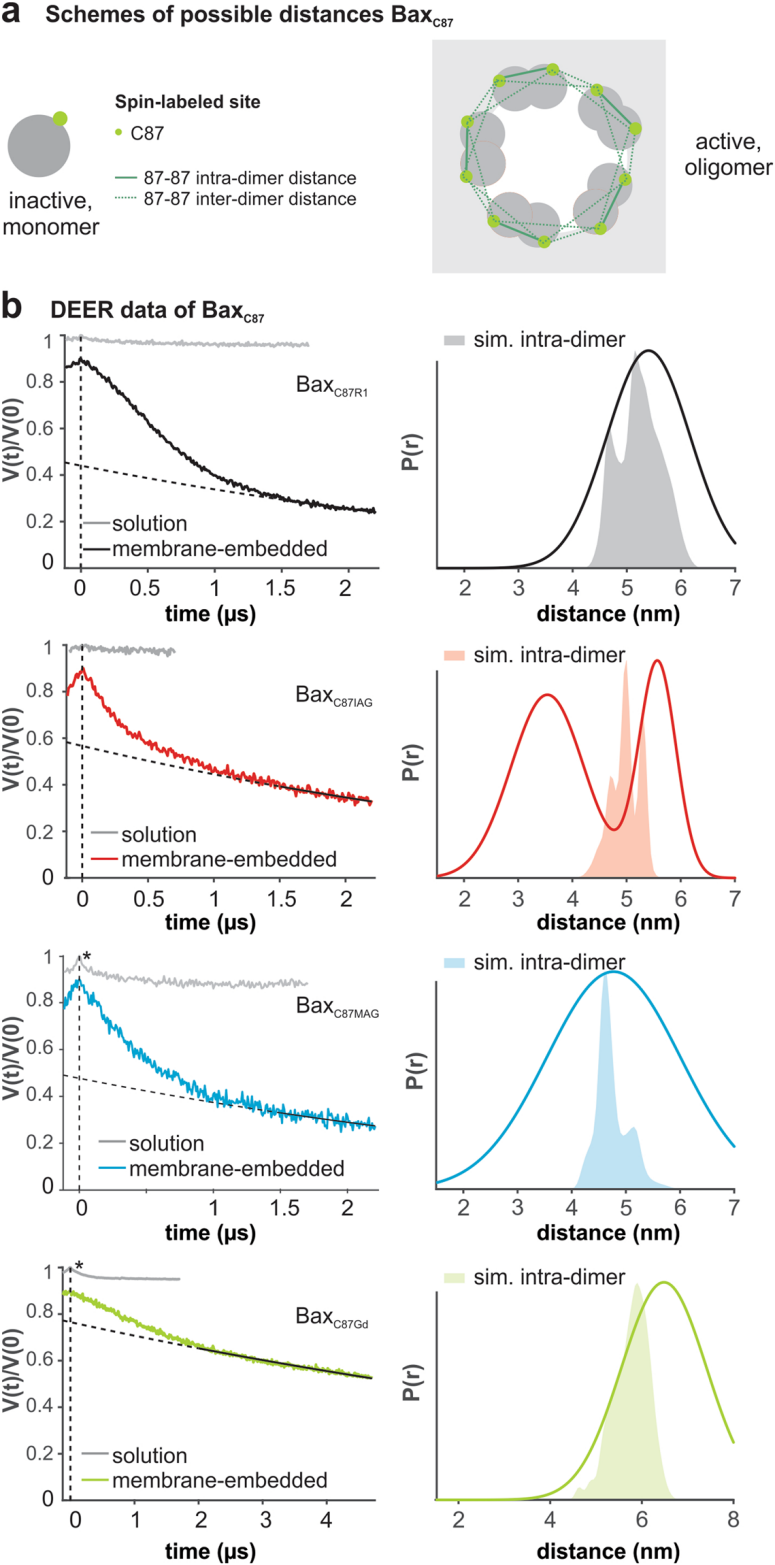
DEER data of spin-labeled Bax_C87_ variants in solution and in membranes. (**a**) Schemes of possible interspin distances. (**b**) Primary data with background functions (left) and distance distributions (right) in the monomeric (solution) and oligomeric (membrane-embedded) conformations obtained by one- or two- Gaussian analysis with DeerAnalysis2018^54^. Shaded areas present simulations of intra-dimer distances based on the structures in^18,20^. The DEER analysis of the monomeric variants is provided in Supp. Fig. 7. Data evaluation and validation is given in Supp. Fig. 8.

Carrying only one spin per monomer, the oligomeric Bax_C87_ variants are easier to be analyzed by DEER (Fig. 3). No distances are expected for the monomeric variants in aqueous solution and, indeed, the primary data showed negligible or only minor dipolar modulations (Fig. 3b). The minor dipolar modulations observed for Bax_C87Gd_ and Bax_C87MAG_ (Fig. 3b, asterisks on the grey traces) suggest a minor (<10%) fraction of dimeric proteins in solution. The short distance extracted could indicate a residual off-pathway conformation called domain-swapped dimer (Supp. Fig. 7) and highlights the sensitivity of DEER to recognize structural heterogeneities.

As already shown in our previous study, the active membrane-embedded Bax_C87R1_ variant is characterized by a mean distance of 5.5 nm, in line with the simulated intra-dimer distance in the structural model, (Fig. 3b and^18^). Since Bax forms oligomers larger than dimers, inter-dimer distances could be present as well, but were not identified within the range of reliable distances (1.5–6 nm) even with longer DEER traces (Supp. Fig. 8). Consequently, the inter-dimer distances have to be either hidden in the distribution peak at 5.5 nm, longer than 6 nm or, alternatively, the oligomers are not unique in size and the heterogeneous inter-dimer distances are indistinguishable from the background function (in line with^24,25,39^). For a better comparison, we decided to record all DEER traces with the same length (2.2 µs) for the nitroxide variants. The limiting factor was the poor signal-to-noise ratio of the IAG and MAG variants due to low protein concentrations and fast relaxation times. Despite the lower reliability of the distance contributions >5 nm, we could prove that for Bax_C87R1_, a Gaussian fit of the short DEER traces provided reliable information on the overall distance distribution, when compared to longer traces (Supp. Fig. 8).

Oligomeric Bax_C87MAG_ showed an unexpectedly broader distribution of distances in its active conformation with respect to the MTSL variant and the simulations. Oligomeric Bax_C87IAG_ revealed a non-negligible fraction of distances <4 nm (Fig. 3b), whose contribution is clearly visible as steeper initial decay in the primary DEER traces. It is tempting to speculate that these short distances represent inter-dimer interactions (in line with data on the protein homolog Bak^40^). Intriguingly, this could imply that the IAG-labeled proteins create more compact oligomers than the MTSL-variants. The Bax_C87Gd_ variant embedded in the membrane did not show indication of short distances in addition to the peak at about 6 nm, which is in good agreement with the simulated intra-dimer distance distribution.

In conclusion, considering the activity assay and the DEER data, Bax_C87Gd_ together with Bax_WTR1_ or Bax_WTIAG_ are the most promising candidates for the planned study of Bax with two ‘colors’. A summary of the results obtained on all Bax variants is given in Supp. Table 1.

### DEER experiments on orthogonally spin-labeled Bax oligomers

**Figure 4:**
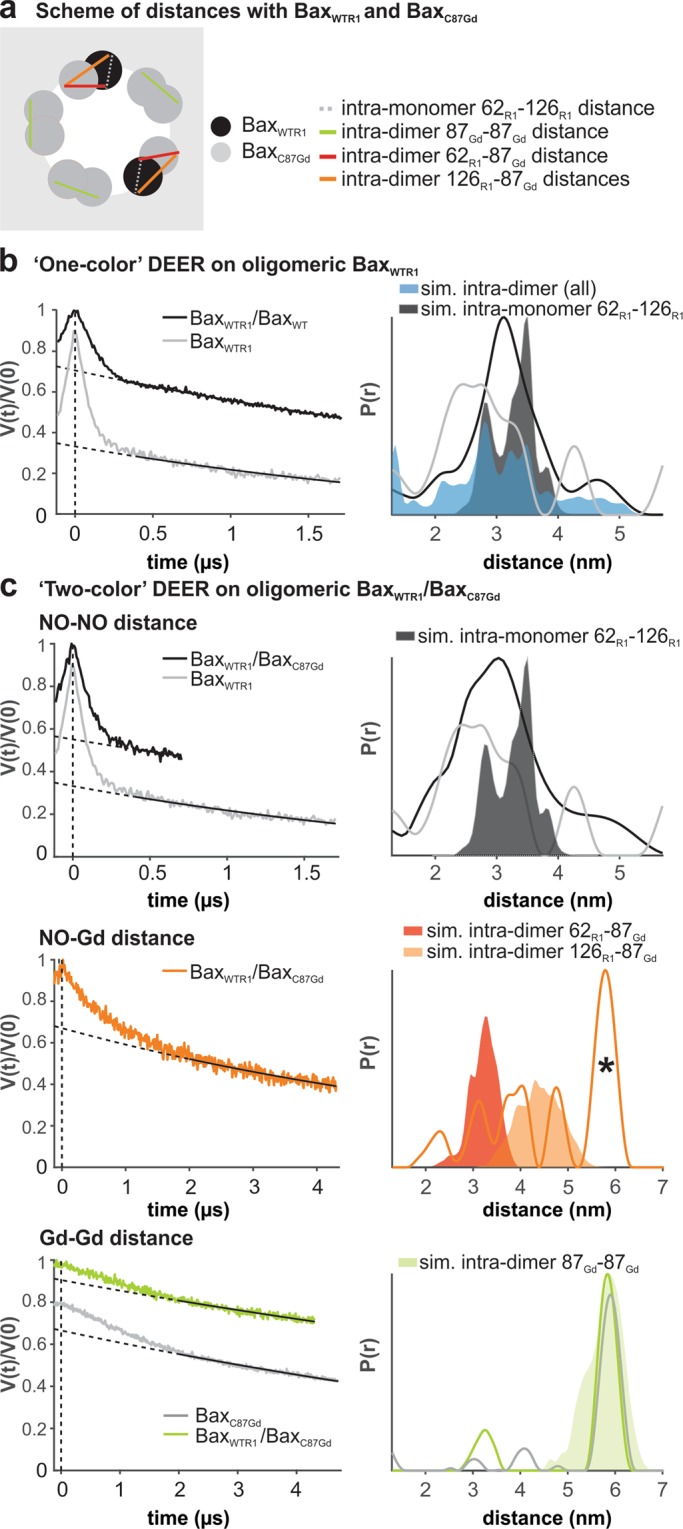
DEER on oligomeric Bax with orthogonal spin labels. (**a**) Scheme of the expected distances**. (b,c**) Primary data with background function (left) and distance distributions (right) obtained by Tikhonov regularization with DeerAnalysis2018^54^. The shaded areas present the corresponding distance simulations based on the structures in^18,20^. (**b**) Active Bax_WTR1_ mixed with 3-fold excess unlabeled Bax_WT_ (black) compared to the undiluted Bax_WTR1_ (grey). (**c**) Upper panel, NO-NO DEER on active Bax_WTR1_ with 3-fold excess of Bax_C87Gd_ compared to the Bax_WTR1_ alone (grey). Central panel, NO-Gd DEER on the same sample. The asterisk highlights a possible channel cross-talk signal. Bottom panel, Gd-Gd DEER on the same sample (green) compared to Bax_C87Gd_ alone (grey). Data evaluation and validation is given in Supp. Fig. 9. The same experiments performed with Bax_WTIAG_ are given in Supp. Figs 10, 11.

The technical novelty of this work is that by mixing Bax_C87Gd_ with Bax_WTR1_ (or Bax_WTIAG_) we can analyze for the first time a complex protein homo-oligomer, which contains NO-NO, NO-Gd, and Gd-Gd distances that can be independently detected with a ‘three-channel’ DEER setup (Fig. 4 and Supp. Figs 9–11). As an additional advantage, by mixing Bax_C87Gd_ in excess with respect to Bax_WTNO_, we also produced an effective nitroxide spin dilution, which statistically enhances the intra-monomeric NO-NO distance in the NO-NO channel, facilitating data analysis. The effect of spin dilution can be observed in a simpler system, in which unlabeled Bax_WT_ was added at 3-fold excess to Bax_WTR1_ before oligomerization (Fig. 4b). The increase in the 3 nm distance fraction in the distribution is in line with the expected enhancement of the intra-monomer distances.

When we mixed Bax_WTR1_ with three-fold excess of Bax_C87Gd_ before oligomerization, we could observe a similar change in the mean distance in the NO-NO channel due to the nitroxide spin dilution. Unfortunately, we could only detect a 0.7 µs trace for the oligomeric active Bax at the membrane due to the fast relaxation of the nitroxide induced by the large amount of gadolinium present in the sample. The smaller change in distance with respect to the dilution with unlabeled Bax_WT_ suggests that the dilution process was less effective (Fig. 4b,c), which is likely due to the slower activation kinetics of Bax_C87Gd_ compared to Bax_WTR1_ (Fig. 1f and Supp. Fig. 12) that might influence the Bax_C87Gd_ to Bax_WTR1_ ratio in the oligomer.

However, on the same sample, we could measure Gd-Gd and Gd-NO distances using the corresponding DEER channels. We detected the expected 6 nm Gd-Gd distance, arising from the interactions between two Bax_C87Gd_ monomers within a dimeric unit (Fig. 4c). Moreover, we identified NO-Gd distances in the 2.5–5 nm range, in line with simulated intra-dimeric 87_Gd_-62_R1_ and 87_Gd_-126_R1_ distances, which confirmed that Bax oligomers containing Bax_C87Gd_ and Bax_WTR1_ monomers are formed (Fig. 4c). Intriguingly, in the NO-Gd DEER channel we also detected a peak at 6 nm (asterisk in Fig. 4c) that is superimposable to the distance detected in the Gd-Gd channel. The latter peak may arise from a residual Gd-Gd contamination in the Gd-NO channel due to the 3-fold molar excess of Bax_C87Gd_ with respect to Bax_WTR1_ (see discussion). We repeated the same experiments mixing Bax_C87Gd_ with Bax_WTIAG_, which provided almost identical results (Supp. Figs 10, 11).

In summary, orthogonal spin labeling experiments on oligomeric Bax were successful and allowed to monitor the formation of Bax oligomers, and to assign on the same sample both intra-and inter-monomer distance peaks, that could not be done with one single ‘color’. This proves the usefulness and applicability of the orthogonal spin labeling of Bax and paves the road towards a DEER-based modeling of membrane-embedded homo and hetero-oligomers of Bcl-2 proteins.

### Spin label biocompatibility in HeLa and *E.coli* cell extracts

As mentioned before, maleimide Gd-DOTA labels are fully biocompatible and MTSL is not stable in cellular context. Up to now, only few data on IAG and MAG labels in cells exist^34–37^. Therefore, we tested and compared the stability of all four labels attached to Bax in a cellular context at concentrations close to the physiological ones. The current lower spin concentration limit for DEER experiments is in the low micromolar range and Bax concentration in human cells was reported to be between 50 and 1000 nM^41^ (500 nM in HeLa cells). Thus, to perform DEER in living cells with acceptable signal-to-noise, supra-physiological Bax concentrations (by a factor 10–20 in HeLa cells) are needed. Notably, this will be the case for almost every protein, as the total protein concentration in mammalian cells was estimated to be 250 mg/ml^42^ and with thousands of different proteins present^43^, the physiological concentration of most proteins will be <1 µM.

**Figure 5:**
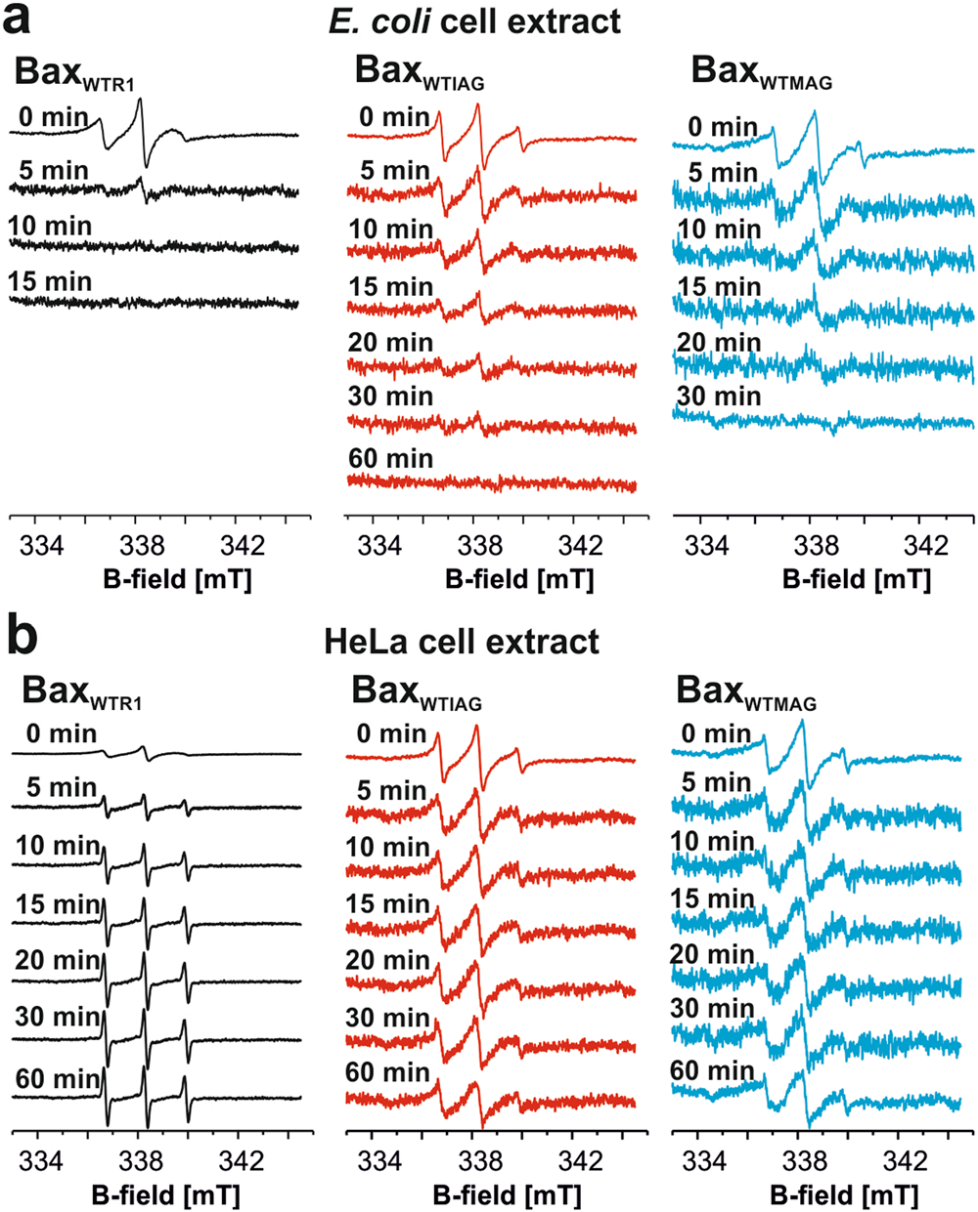
Chemical stability of the nitroxide labels in cell extracts. (**a,b**) cw spectra of Bax_WTR1_, Bax_WTIAG_ and Bax_WTMAG_ after incubation with *E*. *coli* (**a**) or HeLa (**b**) cell extracts. The final cell dilution factor is 6-fold (see Methods). Data on the stability of Gd-DOTA in cell extracts is given in Supp. Fig. 12.

We tested how incubation with diluted *E*. *coli* or HeLa cell extracts affect the spectra of 10 µM Bax_WT_ labeled either with MTSL, IAG or MAG (Fig. 5a,b). The *E*. *coli* extract reduced the radical signal in all probes within 1 h (Fig. 5a), which is consistent with recent data obtained on another spin-labeled apoptotic protein^34^. The NO signal of Bax_WTR1_ disappeared within 10 min, while the signals from Bax_WTIAG_ and Bax_WTMAG_ persisted for a longer time. Notably, the Gd signal intensity of 20 µM Bax_WTGd_ remained unchanged after 1 h incubation with *E*. *coli* extracts (Supp. Fig. 12). Thus, we concluded that spin-labeled recombinant proteins inserted into *E*. *coli* cells at 10–20 µM concentrations can only be analyzed by DEER if Gd-based labels are used. To monitor via DEER nitroxide-labeled proteins in *E*. *coli* at physiologically relevant concentrations, biochemical tricks like freezing the cells immediately after protein addition, using cells with fewer reducing agents, or probing proteins on the outer surface of the outer cell membrane are necessary^44,45^.

Surprisingly, in HeLa cell extracts, nitroxide labels attached to Bax were found to be more stable (Fig. 5b), highlighting the different chemical components present in these cells. IAG and MAG labels showed no detectable signal reduction upon 1 h of incubation, while MTSL was released from the protein within 30 min, and the NO group was partially chemically reduced. As expected, the maleimide-Gd-DOTA signal was not affected by incubation with HeLa cells (Supp. Fig. 12). This proves that in-cell nitroxide-gadolinium DEER experiments at protein concentrations close to physiological ones might be possible in HeLa cells.

### Protein subcellular localization

The first requirement for in-cell EPR is that the cells survive the protein transfer process to ensure that ‘living’ healthy cells are frozen for the DEER experiments. Every mechanism transporting a folded protein into a cell will affect the integrity of the outer membrane and likely kill a fraction of the cell population. Consequently, it is desirable to be able to remove dead cells before insertion in the DEER tube. For this reason, we first chose to use osmotic shock on surface-attached HeLa cells as “protein transfer mechanism”. Dead cells detach and are removed together with the residual non-incorporated proteins by washing steps.

**Figure 6:**
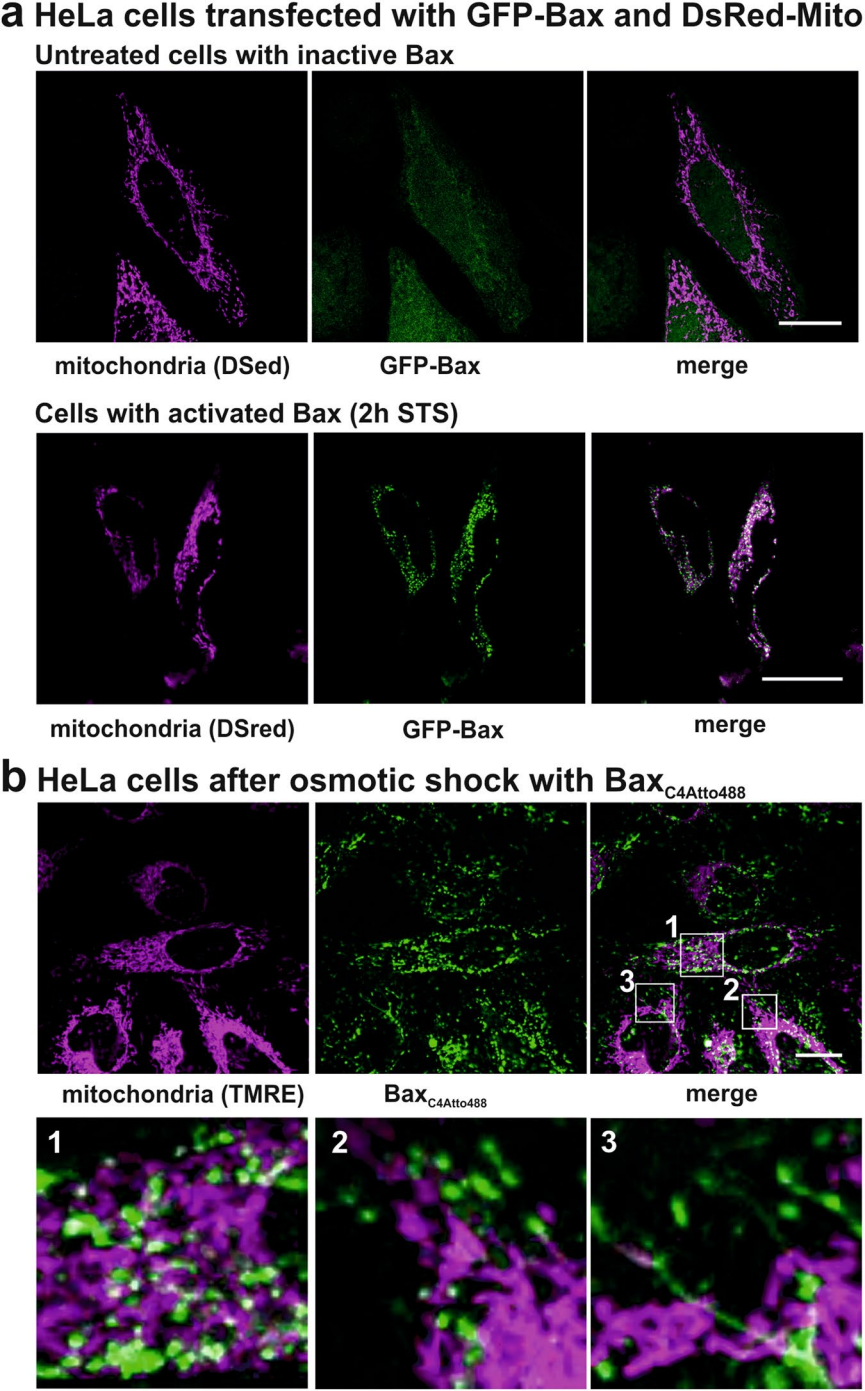
Comparison of the localization of Bax expressed in cells or transferred by osmotic shock. (**a**) HeLa cells transfected with plasmids for the mitochondrial marker DsRed-Mito (magenta) and GFP-Bax (green) before and after pro-apoptotic stimuli (1 µM Staurosporin, STS). (**b**) HeLa cells after osmotic shock, showing the uptake of Bax_C4Atto488_ (green). To visualize the mitochondria and their integrity, TMRE (magenta) was added as mitochondrial marker. Upper row: overview images; lower row: enlarged sections. Scalebar: 20 µm. Additional control experiments are given in Supp. Fig. 13.

Another essential requirement is that the transferred folded protein is correctly localized within the cell, so that the physiological interaction partners and the native microenvironment are present. As described in the introduction, Bax is mainly cytosolic in healthy cells, and it translocates to the mitochondrial outer membrane upon pro-apoptotic stimuli. Using the transfection method we were able to observe this localization in HeLa cells using GFP-Bax (Fig. 6a). However, for EPR studies we would need to transfer recombinantly-produced spin-labeled Bax into cells. Towards this end, we were able to transfer folded, monomeric Bax_Atto488_^19,46^ into HeLa cells by applying an osmotic shock. We found that many cells survived the transfer process. Unfortunately, Bax was not localized in the cytosol (Fig. 6b), but in small intra-cellular compartments, which were not mitochondria as addressed by TMRE staining. Additionally, TMRE showed that the mitochondria kept their potential and were therefore not permeabilized by Bax. Thus, Bax_Atto488_ was taken up by the cells but not correctly localized.

As a control we transferred a fluorescently labeled Dextran into the HeLa cells, which, as expected, was mostly localized in the cytosol (Supp. Fig. 13). Interestingly, in a minor cell population the Dextran was detected in similar compartments as Bax_Atto488_ and a similar localization pattern was observed for fluorescent ubiquitin in HeLa cell after osmotic shock^32^. Thus, protein mislocalization might be a common problem after protein transfer using osmotic shock. We surmise that HeLa cells recognize the recombinantly-produced Bax as a non-correctly folded, surplus or pathogenic protein, and start recycling (degradation) pathways like ERAD (endoplasmatic-reticulum-associated degradation) or autophagy to remove it.

We tried to overcome this problem by using another protein transfer technique such as electroporation, but we did not achieve a significant protein transfer yet. However, our work shows that protein mislocalization in cells is an issue and all future in cell EPR studies should carefully address protein localization. Moreover, it shows that recombinant proteins are not homogenously distributed in the target cells. In fact, we found cells containing high or low protein concentrations, and some cells showed undetectable amounts of recombinant proteins. Possibly, the use of isolated mitochondria may offer an alternative successful approach to investigate Bax oligomers under native membrane environments, avoiding the transfer process.

## Discussion

This study systematically addresses challenges and advantages of using DEER and orthogonal spin probes on homo-oligomers of Bax at the membrane and highlights potentials and pitfalls of in-cell DEER studies at protein concentrations close to the physiological ones.

We used four different spin probes on two Bax variants and could identify that specific labels at selected sites can slow down pore formation, enhance Bax auto-activation or induce misfolding. Thus, protein fold and activity can be affected by labeling (as well as by point mutations) and should always be tested prior to structural studies. We found that Bax_WTR1_, Bax_WTIAG_, Bax_C87R1_ and Bax_C87Gd_ were well folded and active based on pore-forming assays and DEER data, while the MAG label was not well tolerated by Bax.

Overall, the orthogonal spin labeling approach in combination with ‘two-color-three-channel’ DEER was successful on Bax homo-oligomers at the membrane and enabled us to decipher intra- and inter-monomer distances in a complex multi-spin system. These results open a new way towards EPR structural analysis of membrane-embedded Bcl-2 homo- and hetero-oligomers.

By comparing the DEER data obtained with different labels, we found that oligomeric Bax_C87R1_ and Bax_C87Gd_ showed no distances shorter than 4 nm, while Bax_C87IAG_ and Bax_C87MAG_ did. The short distances could be a hint for inter-dimer interactions, which could not be detected by EPR in Bax oligomers up to now. Interestingly, similar distances were already detected on spin-labeled C- and N-terminally truncated Bak oligomers at protein concentrations higher than those used here^40^. It is possible that the attachment of some labels to Bax as well as the missing protein termini in Bak can affect the labile inter-dimer contacts^23,24,47^ within the oligomers and thereby modify the size and homogeneity of the oligomers. Alternatively, some labels may populate different rotamers due to steric hindrance or interactions with the membrane bilayer, which would affect the measured distance distributions.

Interestingly, our experiments on orthogonally-labeled Bax oligomers identified an unexpected peak at 6 nm in the NO-Gd channel which was similar to the one detected in the Gd-Gd channel on the same sample. There are three possible explanations for this peak. First, it could be an artifact due to the short length of the DEER time trace. Second, there could be additional inter-dimeric 87_Gd_-62_R1_ and 87_Gd_-126_R1_ distances in the 6 nm range within the oligomer that cannot be predicted as the available model is limited to a dimer. Third, there might be a channel cross-talk, which means that residual Gd-Gd distances can be detected in the NO-Gd channel (already addressed in a simpler spin system in^48^), since NO and Gd are not perfectly spectroscopically orthogonal at Q band. Notably, the extent of such cross-talk signals can be strongly dependent on the complexity of the spin systems under study and on the excess of gadolinium versus nitroxide labels. A more systematic study is necessary to address the impact of non-perfect orthogonality when complex multi-spin oligomers are present with different ratios of the two ‘colors’.

Another relevant aspect addressed here is the stability of the four labels in cellular context at spin concentrations of a few micromolar. This study reveals that only the Gd-DOTA label can withstand the conditions in *E*. *coli* cells, therefore orthogonal strategies in *E*. *coli* cells at physiological concentrations are not yet possible. In contrast, IAG and MAG are resistant in HeLa cell extracts, which opens the possibility to use ‘two-color’ DEER with gadolinium and nitroxide labels in HeLa cells.

The cell imaging experiments revealed two additional general problems to be considered for in-cell DEER: first the difficulties of a recombinant proteins to reach the physiological localization of the endogenous protein in the cell; second, the non-homogeneous distribution of proteins in different cells. Non-apoptotic proteins may have an easier access to their correct compartment; however, it will be always challenging to reach homogenous and reproducible concentrations of the probe inside different cells. The concentration of the transferred protein will vary between cells and sub-compartments, which will create steep and unpredictable DEER background signals, as already visible in the published in-cell DEER traces with gadolinium labels^32,44,45^.

We are at the beginning of an exciting journey to explore the overwhelming complexity of proteins’ structure and interaction in cellular environments by EPR and to build coarse-grained structural models of hetero- and homo-oligomeric membrane-embedded Bcl-2 protein complexes at the mitochondrial outer membrane at the onset of apoptosis.

## Methods

### Protein production and labeling

Full length mouse Bid, cBid, and full length human Bax were expressed in *E*. *coli* and purified, and in case of cBid cleaved, as described in^30,49^. Bax mutants were produced by site-directed mutagenesis and purified in the same way as the wild type protein. The mutants used here were already introduced in^18^. Bax (15–20 µM) was labeled with (1-Oxyl-2,2,5,5-tetramethyl-∆3-pyrroline-3-methyl) methanethiolsulfonate (MTSL, Toronto Research Chemicals), iodacetamide *gem*-diethyl nitroxide (IAG^34^), maleimide *gem*-diethyl nitroxide (MAG^34^) and maleimide Gd-DOTA (Gd, Macrocyclics). Stock solutions of the nitroxide labels were prepared in DMSO, of the maleimide Gd-DOTA in buffer (pH 6.8, 500 mM NaCl, 150 mM MOPS, 10% v/v glycerol). Labels were added on ice in a 5–8 fold excess and samples were incubated overnight at 6–8 °C. Free label was removed using Vivaspin turbo 4 concentrators (5.000 MWCO, Sartorius, Germany). The final protein concentration was calculated based on UV spectra with a molar extinction coefficient of 36940 M^−1^cm^−1^ for Bax and 8490 M^−1^cm^−1^ for cBid. The spin concentration of nitroxide labeled Bax was calculated comparing the double integral of the X-band cw spectra to that of a TEMPOL 100 µM stock solution. The spin concentration of Gd-DOTA labeled Bax was estimated based on the comparison of the intensity of the Q-band field swept spectra at 10 K with that obtained on a soluble maleimide Gd-DOTA of known concentration (Supp. Fig. 2).

### Composition of the lipid mixtures and LUV preparation

The lipid mixture mimicking the MOM composition was prepared as in^18,22^ with 46% egg L-α-phosphatidyl choline (PC), 25% egg L-α phosphatidyl ethanolamine (PE), 11% bovine liver L-α-phosphatidyl inositol (PI), 10% 18:1 phosphatidyl serine (PS) and 8% cardiolipin (CL) (all percentages w/w). Lipids were mixed in chloroform, dried under vacuum and flushed with nitrogen or argon and stored at −28 °C. To form LUV´s for DEER experiments the dried lipid mixture was dissolved in buffer (150 mM NaCl, 20 mM TRIS pH 7.5). For vesicle content release assays a different buffer was used (see below). Afterwards, the solution underwent five cycles of freezing and thawing followed by liposome extrusion, in which the liposome solution was passed 31 times through a membrane (400 nm pore size).

### Vesicle content release assays

The dried lipid mixture mimicking the MOM composition was dissolved in 80 mM calcein (pH 7.0; Sigma) followed by five cycles of freezing and thawing and 31 passages through a membrane (400 nm pore size). To remove the non-entrapped fluorophore, vesicles were dialyzed again with buffer (140 mM NaCl, 20 mM HEPES, 1 mM EDTA; pH 7.0) followed by one passage through a PD-10 column (Biorad, Hercules CA). The success of the procedure was tested by comparing the fluorescence signal of the vesicles before and after addition of 0.2% Triton, which destroys the membrane and releases all fluorophores. Vesicles were only used for content release assays when Triton addition raises the fluorescence intensity (ex: 495 nm, em: 520 nm) ≥7-fold. Kinetic experiments were done at 37 °C using cuvettes that can be stirred (high precision cell 109000F-10–40 from Hellma Analytics Müllheim, Germany) in a JASCO Spectrofluorometer 8500 (JASCO, Pfungstadt, Germany) equipped with an 8-fold cuvette exchanger and a temperature control unit. Samples were measured over 60 min taking data points every 30 s. At the beginning of the kinetic curve the cuvettes contained only buffer and vesicles. The proteins were added after ≥3 data points at the protein concentration indicated in the figure (lipid to Bax ratio: ~500 to 1). Changes in the fluorescence intensity were analyzed over time as: F_t_^N^ = (F_t_-F_0_)/(F_Triton_-F_0_), where F_t_^N^ is the normalized fluorescence intensity at time t, F_t_ is the measured fluorescence intensity at time t, F_0_ is the measured fluorescence intensity at time zero before protein addition and F_Triton_ is the measured fluorescence intensity after Triton addition at the end of the measurement. Proteins and Triton are added from stock solutions at high concentration, so that the addition negligibly affects the sample volume.

The rather high standard deviation (SD) for Bax-induced pore formation is an intrinsic property of these experiments that require addition of many protein-lipid components. The absolute values of the pore-forming activity depend on many variables as the lipid or protein batch, the exact degree of spin labeling, the liposome preparation and the protein dilution. For example, if the membrane for the extrusion process is not perfect, the fraction of multilamellar vesicles can increase. As Bax cannot easily reach the inner lipid shell, the maximally reached permeabilization by Bax (but not by the detergent) is reduced. However, based on >20 vesicle content release assays we calculated mean and SD for 50 nM unlabeled Bax_WT_ in absence (mean: 22; SD: 12) or presence (mean: 83; SD: 10) of 50 nM cBid. An unpaired two-tailed t-test revealed that the difference between the two conditions is statistically extremely significant (p: < 0.0001) and thus the complete data set produces consistent data. For all labeled protein the situation is even more complex as different protein batches are shown that will slightly vary in the labeling efficiency (mostly +/− 10%), which can increase the SD, especially in case labeling reduces or abolishes the activity while the residual unlabeled protein will still form pores.

### EPR: Continuous wave spectroscopy

All cw EPR experiments were performed at room temperature on samples of 20 µl volume in 1.5 mm outer diameter glass tubes with an X-band MiniScope MS 5000 (Magnettech by Freiberg Instruments; Freiberg; Germany) spectrometer. The spectra were acquired using 10 mW microwave power, a modulation amplitude of 0.1 mT at 100 kHz modulation frequency, a sweep width of 15 mT at 80 s sweep time.

### EPR: Double electron-electron resonance spectroscopy

The DEER samples of the inactive, monomeric Bax in buffer contained 50% v/v glycerol-d_8_ as cryoprotectant. For the active, membrane-embedded, oligomeric samples, inactive Bax was mixed with cBid at a molar ratio of 1 to 0.5–1 and liposomes mimicking the MOM, with 500 lipids per Bax monomer. Samples were incubated for 2 h at 37 °C before centrifugation at 120,000 g for 30 min (Airfuge; Beckman Coulter; Brea; CA). DEER measurements were performed on the pellet fractions after addition of 16 µl buffer and 10% v/v glycerol-d_8_ with a final Bax concentration of 15–30 µM. The samples were inserted in 3 mm outer diameter quartz tubes and flash frozen in liquid nitrogen.

All pulsed EPR experiments were performed with a Bruker Q-band Elexsys E580 SpinJet-AWG spectrometer equipped with a 150 W TWT amplifier and a home-built resonator accomodating 3 mm tubes. The DEER experiments were performed using the dead-time free 4-pulse DEER sequence (*π* /2)_obs_-(d1)-(*π*)_obs_-(d1 + T)-(*π*)_pump_-(d2-T)-(*π*)_obs_-(d2)-(echo)^50,51^ with 16-step phase cycling^52^. The main frequency of the microwave bridge was fixed at the observer position and the offset of the pump frequency was synthesized by the AWG. All experiments were performed using Gaussian pulse shapes with the following parameters: for NO-NO (at 50 K) and Gd-Gd (10 K) DEER experiments we used 13.6 ns FWHM (32 ns time base)^53^ at 100 MHz separation between pump and observer. NO-Gd experiments (10 K) were performed at 280 MHz separation, using the same type of Gaussian observer pulses in combination with a 10.2 ns FWHM (24 ns time base) pump pulse. The schematic of the three setups used are shown in Supp. Fig. 15. The data were analyzed using DeerAnalysis version 2018^54^ and all simulations of DEER distance distributions were obtained by MMM Version 2018.2^55^.

### Preparation of HeLa and *E. coli* cell extracts and test of label reduction

*E*. *coli* cell extracts were prepared as follows: 5 l BL21/RIPL/pTYB1-Bax cells (BL21/RIPL cells from Agilent; Waldbronn; Germany) were grown for Bax expression as explained in^23,49^. Cells were harvested by centrifugation at 6,000 rpm for 20 min in an Avanti JXN-26 centrifuge with JLA-8.1000 rotor (Beckman Coulter; Brea; CA). Cell pellets were transferred into 50 ml tubes and suspended in buffer (150 mM NaCl, 20 mM TRIS; pH 7.5). Thereby, the buffer volume was set to be 1–2 times the volume of the cell pellet. To break the membranes, cells were passed 3 to 5 times through an Emulsiflex C5 at high pressure following the manufacturer’s instructions (Avestin; Mannheim; Germany). Afterwards, the cells were incubated 30 min on ice with DNase I (Roche; Mannheim; Germany) and then centrifuged for 1 h at 4 °C and 25,000 rpm in an Avanti JXN-26 centrifuge with a JA-25.50 rotor (Beckman Coulter; Brea; CA) to remove membranes and non-disrupted cells. Based on the volume of the cell pellet, the supernatant after centrifugation represents a ~3-fold diluted *E*. *coli* cell extract, which was aliquoted, shock frozen in liquid nitrogen, and stored at −30 °C before use.

HeLa cell extracts were prepared as follows: 20–35 * 10^6^ HeLa cells were centrifuged at 1000 g for 5 min at RT, washed with PBS followed by a second centrifugation step (1000 g for 5 min at RT). Each HeLa cell has a volume of ~1000–5000 µm³^56,57^. Using an average volume of 3000 µm³, 20–35 * 10^6^ cells will have a volume of 60–120 µl. Therefore, we suspended the cell pellet in 50–100 µl of a 10% octylglucoside solution (Anatrace; Maumee; OH). Afterwards, the solution was spun down at 20,000 g for 10 min at 10 °C and the supernatant used as ~3-fold diluted *HeLa* cell extract. The extract was shock frozen in liquid nitrogen and stored at −30 °C. To test the effect of the diluted cell extracts on the spin labeled proteins, we mixed 10 µl Bax_WTR1_, Bax_WTIAG_ or Bax_WTMAG_ with 10 µl *E*. *coli* or *HeLa* cell extract to a final protein concentration of 8–12 µM and X-band cw EPR spectra were measured at room temperature.

### Experiments with living HeLa cells

HeLa cells were maintained in Dulbecco’s modified Eagle’s medium (DMEM) supplemented with 10% heat-inactivated fetal calf serum (FCS) (Invitrogen; Carlsbad; CA) and 1% penicillin/streptomycin in 5% CO_2_ at 37 °C. For experiments based on DNA transfections, cells were seeded and transfected with Lipofectamine 2000 (Invitrogen) with 100 ng of GFP-Bax and 100 nM pDS-Red-Mito (Clontech; Mountain View; CA) according to the manufacturer’s instructions. Cells were maintained at 37 °C and 5% CO_2_ on DMEM without phenol red supplemented with FCS. To induce apoptosis 1 mM staurosporine was added.

For osmotic shock experiments ~8000 HeLa cells were seeded per well in 8 well Nunc Lab-Tek chambers and incubated overnight in DMEM supplemented with 10% heat-inactivated FCS and 1% penicillin/streptomycin in 5% CO_2_ at 37 °C. The cells were washed with PBS buffer and then incubated with the protein or sugar of interest in 0.3 fold PBS for 45 min at 37 °C. Afterwards, the medium was exchanged to DMEM (supplemented as mentioned above) and the cells were incubated for 1 h at 37 °C before 50 nM TMRE was added to the medium and the cells incubated for another 15 min at 37 °C. Then the cells were washed 4 times with PBS and afterwards placed in DMEM without phenol red supplemented with FCS for direct imaging.

Cell imaging experiments were performed on a Zeiss LSM 710 microscope at 37 °C using a C-Apochromat 40× N.A. 1.2 water immersion objective (Zeiss; Jena; Germany). Image preparation was done using Fiji^58^.

## Supplementary information

Supplementary figures and Table
