# Supplementary material for: A new perspective on membrane-embedded Bax oligomers using DEER and bioresistant orthogonal spin labels
Source: Sci Rep. 2019 Sep 10;9:13013. doi: 10.1038/s41598-019-49370-z (PMC6737250; doi:10.1038/s41598-019-49370-z)
Supplement: Supplementary file 1 — Supplementary figures and Table [file 41598_2019_49370_MOESM1_ESM.docx]

*Supplementary Information*

A new perspective on membrane-embedded Bax oligomers using DEER and bioresistant orthogonal spin labels

Markus Teucher, Hui Zhang, Verian Bader, Konstanze F. Winklhofer, Ana J. García-Sáez, Andrzej Rajca, Stephanie Bleicken and Enrica Bordignon

|  | **Bax_WT_** | **Bax_C87 (C62S, C126S)_** |
| --- | --- | --- |
| **MTSL** | ☺   - activity indistinguishable from Bax_WT_ - correctly folded in both conformations | ☺   - increased auto-activity - correctly folded in the active conformation - no or very few dimers in solution |
| **IAG** | ☺   - increased auto-activity - correctly folded in both conformations | ☺   - auto-activity similar to Bax_WT_ - mildly reduced activity in presence of cBid - correctly folded in the active conformation (additional distance in the oligomeric form that might indicate inter-dimer contacts) - no or very few dimers in solution |
| **MAG** | 😐   - increased auto-activity - correctly folded in both conformations - increased protein aggregation propensity | ☹   - auto-active protein - active conformation with a broader distance distribution than the other variants, which might indicate partial protein aggregation - increased protein aggregation propensity - detectable fraction of dimers in solution |
| **Gd** | ☹   - low activity - not correctly folded | ☺   - very low auto-activity - lower activity than Bax_WT_ - correctly folded in the active conformation - detectable fraction of dimers in solution |

**Supplemental Table 1: Summary of the results obtained from the different spin-labeled Bax variants.**


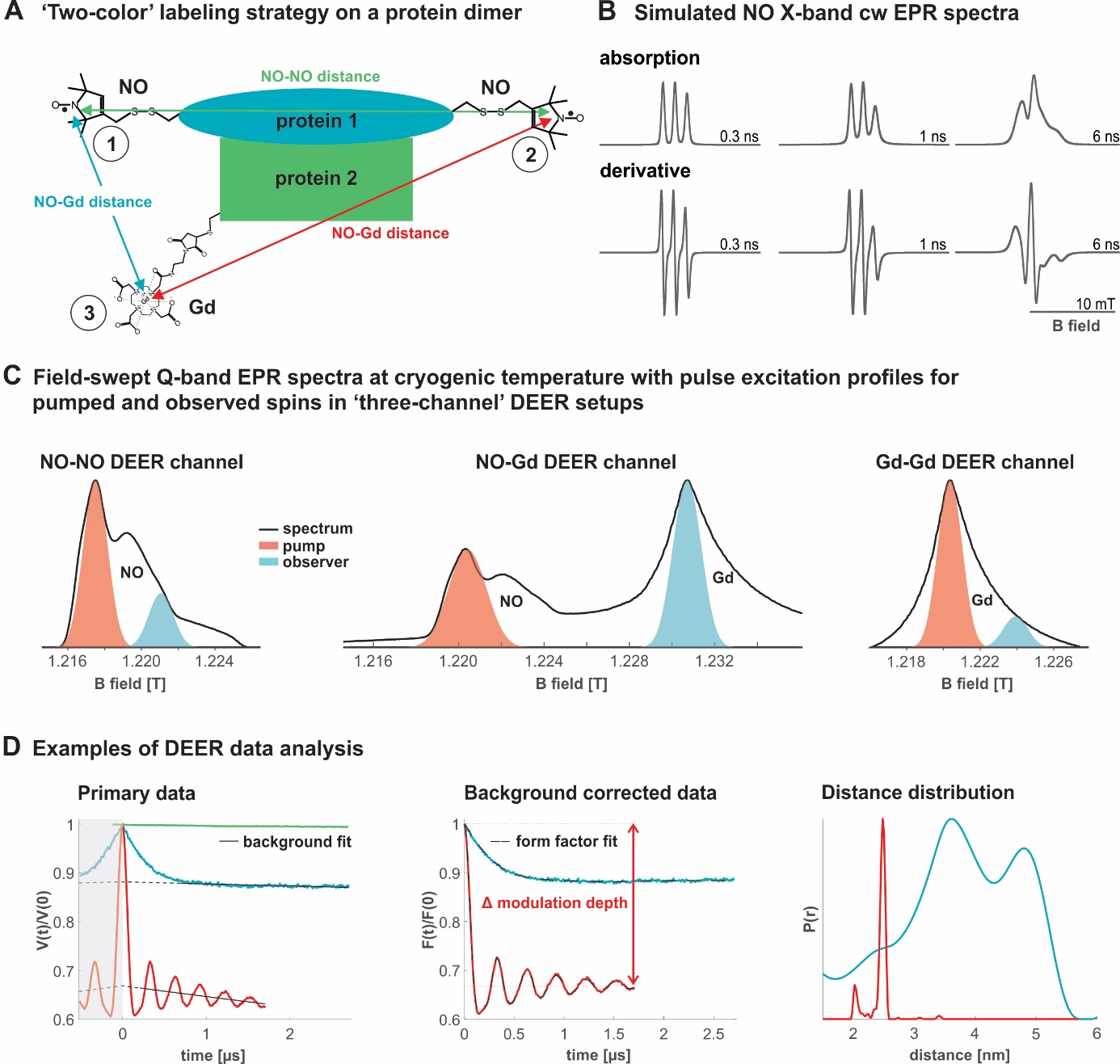


**Supplemental Figure 1: DEER in a nutshell.**

**A)** Schematic view of a spin-labeled protein which illustrates the usefulness of ‘two-color-three-channel’ DEER. The dimer contains two nitroxide spin labels (NO) bound to one protein and one gadolinium spin label (Gd) bound to the second. All measurable inter-spin distances (1-2, 1-3, 2-3) are indicated by arrows. With three labels, we can detect in general three different distances. In a conventional approach using one type of spin label, all distances are detected simultaneously and their assignment in the overall distance distribution is challenging. By using ‘two-color-three-channel’ DEER we can reduce the complexity as distance 1-2 will be only visible in the NO-NO DEER channel, no distance will appear in the Gd-Gd channel, while distances 1-3 and 2-3 will be detectable in the NO-Gd channel.

**B)** Nitroxide (NO) spin labels can be detected via continuous wave (cw) EPR at room temperature. Their spectral shapes provide information about the dynamics of the bound label, which is modulated by its microenvironment. This is illustrated via three simulated nitroxide cw EPR spectra (absorption and derivative signal) with different rotational correlation times representing a very mobile (left), an intermediate (center) and an immobile (right) nitroxide probe attached to a protein (simulated with EasySpin^1^). In cw EPR, spectra are recorded in their derivative form to maximize signal-to-noise ratio. The area under the absorption spectra is used to determine the spin concentration, thereby the labeling efficiency if the protein concentration is known. The cw spectrum of Gd-DOTA spin labels is below detection limit at < 200 micromolar concentration at X-band.

**C)** Field-swept Q-band EPR spectra of the nitroxide and Gd-DOTA labels (black) detected at cryogenic temperatures. In pulsed EPR, absorption spectra are recorded. Notably, the two labels are spectrally distinct but not fully orthogonal because they have some spectral overlap. To perform DEER experiments, one fraction of the spectrum is excited/pumped (indicated by the red area) and another fraction is observed (observer, blue area). The pulse excitation profiles are simulated with EasySpin^1^. Notably, due to the large width of the EPR spectra of both nitroxide and gadolinium, even when the two interacting spins are of the same type (e.g. monomeric Bax_WT_ spin labeled at sites 62 and 126 with MTSL), one fraction of the labels at site 62 or 126 is excited and another fraction of labels at site 62 or 126 is pumped, allowing detection of distances between interacting spins (62-126) of the same type (which can be related to homo-FRET experiments). The pump/observer excitation schemes used for the NO-NO, NO-Gd and Gd-Gd channel are shown.

**D)** We perform DEER experiments using the 4-pulse DEER sequence, reviewed in^2,2^ with Gaussian pulses^3^. In a DEER experiment we record the pump-induced modulation of the observer signal versus time in a primary time trace (called V(t) in the left panel). The modulation is induced by the existing interaction (called dipolar coupling, which can be expressed in energy or frequency units) between the two magnetic dipole moments associated with the two spins. The dipolar frequencies detected in the primary trace encode the information about the distances between the pump and observer spins. The primary data show not only the intra-complex distances of interest, but additional inter-complex distances that create a “background function”, and this background depends on the protein concentration (the lower the concentration, the less relevant is the background) and the spatial distributions of the spins (soluble protein in general have a mono-exponential background function, and proteins embedded in membranes may have different background functions). This background function needs to be fitted and removed from the primary data in order to obtain the so-called form factor (middle panel) from which the distance distribution can be extracted. Different methods exist to determine the distance distribution, the most commonly used are Tikhonov regularization or Gaussian fit, as implemented in the DeerAnalysis software^2,3^. Three examples of DEER data are shown, to highlight important features. The green time trace is recorded on a monomeric protein with only one label. It therefore shows a pure background decay function without dipolar frequency, because there are no specific interactions between the spins in the sample. The red and blue time traces show dipolar oscillations, indicating that the spins in the sample are interacting specifically. In the red trace we clearly identify the dipolar oscillations, which means that we have a narrow distance distribution. In contrast, in the blue trace there is an interference of many dipolar frequencies, that dampens the frequency modulation of the time trace and indicates a broad distance distribution. Finally, we can extract the so-called modulation depth from the form factors (middle panel). The modulation depth depends on the degree of spin labeling of the protein and when studying a protein complex, on the fraction of interacting spin-labeled proteins. The higher the modulation depth the higher the labeling efficiency and/or the fraction of interacting spins in the sample. Notably, in oligomeric proteins, multispin distances are detected, which also increases the overall modulation depth.

**
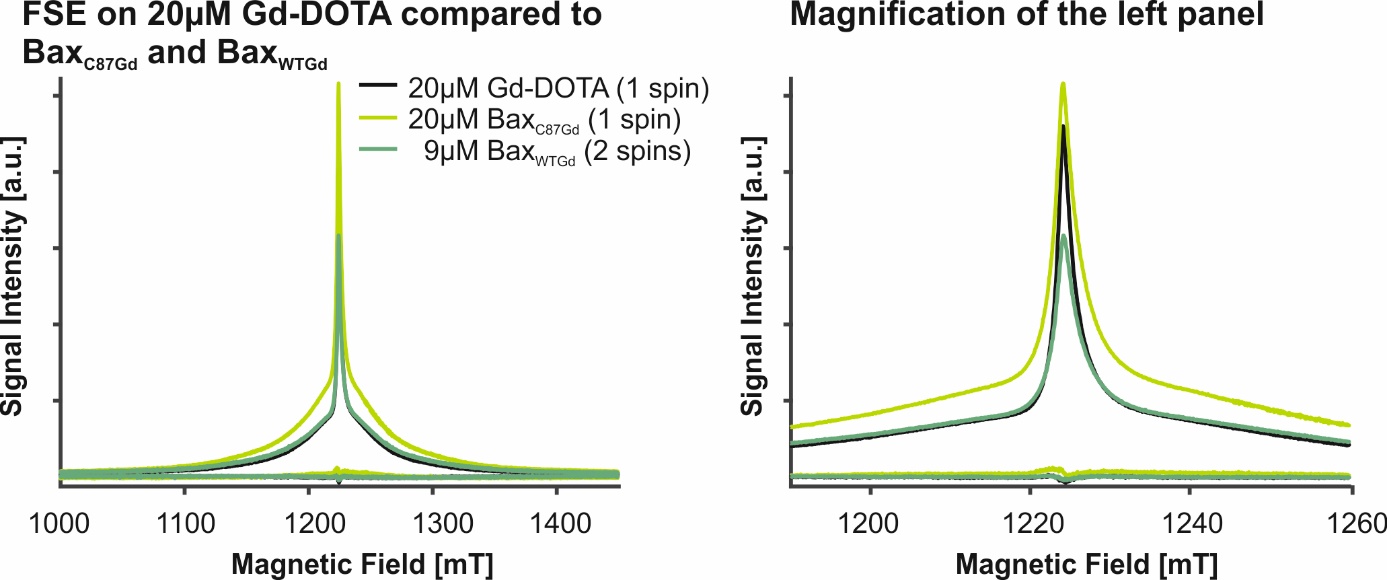
**

**Supplemental Figure 2 (related to Figure 1c): Estimation of the spin concentration of the Gd-labeled Bax variants.** Q-band field swept echo (FSE) spectra acquired at 10 K using Gaussian pulses^4^. The microwave frequency is set to the center of the resonator dip. A 16-400-32 ns Hahn echo sequence is used with srt = 2000 µs, 10,000 points, 450 mT sweep width, n=1, h=100, video gain 6 dB. The three samples contain: 20 µM Gd^3+^-DOTA in solution, 20 µM Bax_C87Gd_ carrying maximally one label and 9 µM Bax_WTGd_ carrying maximally two labels. Both real and imaginary part of the spectra are shown. The right panel shows a magnification of the central region of the FSE spectra. Normalized to the Gd-DOTA reference, the maximum amplitudes of the FSE spectra are 1.13 for Bax_C87Gd_ (corresponding to 22 µM spin, 110% labeling efficiency) and 0.68 for Bax_WTGd_ (14 µM spin, 80% labeling efficiency) with errors estimated to be in the order of 10-20%. We can conservatively state that the labeling efficiency of both Bax mutants is >50%.

**
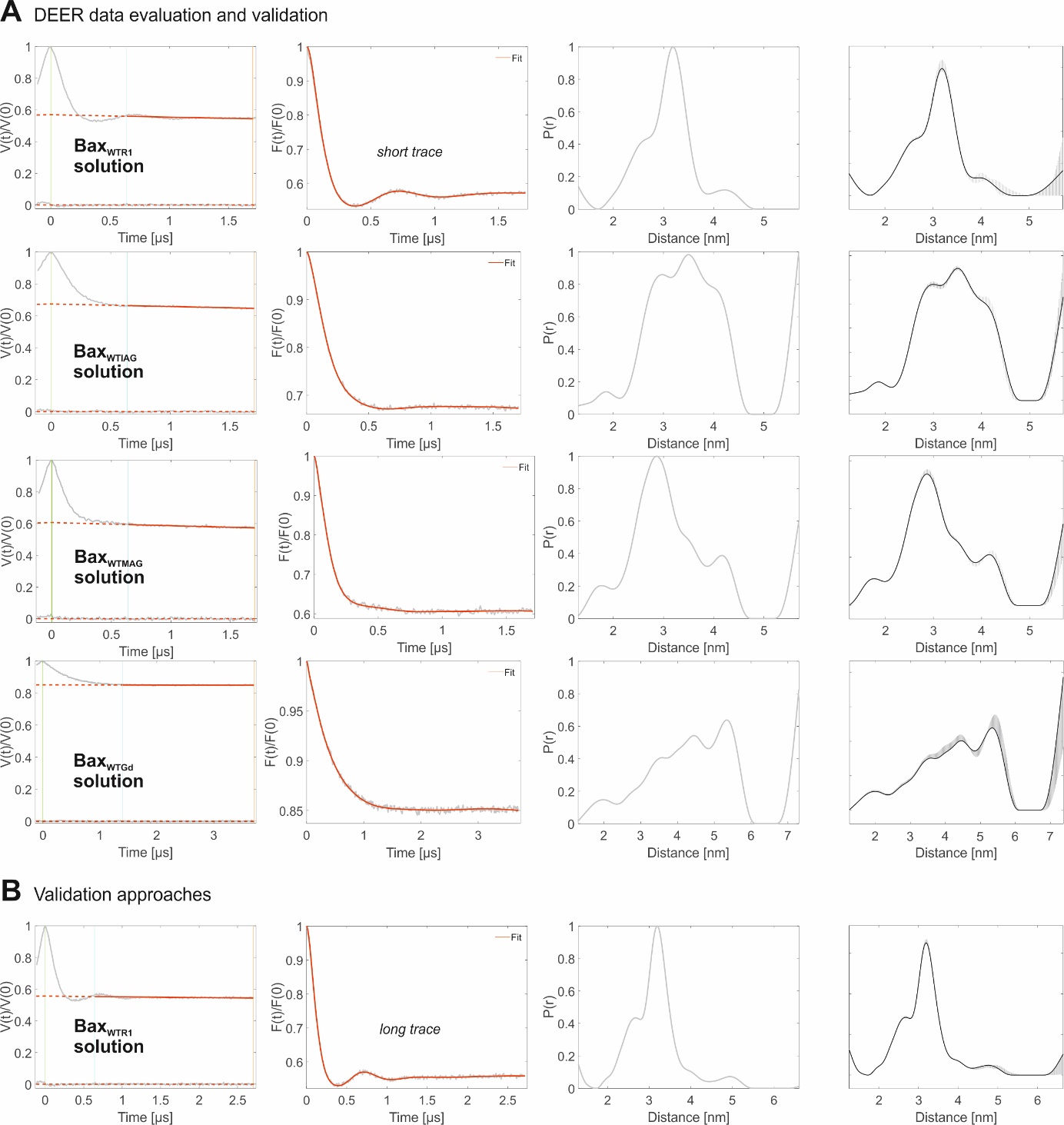
**

**Supplemental Figure 3 (related to Figure 2): DEER data evaluation and validation of the monomeric Bax_WT_ variants in solution. A-B)** Deer data evaluation and validation performed with DeerAnalysis2018^3^. From left to right: primary DEER traces with background fit; form factors with fit; obtained distance distributions (Tikhonov regularization parameter 100 for NO and 1000 for Gd). Color code as in Figure 2b. Fits are shown as red lines; dotted regions are excluded from fitting. Right panels: Data validation performed by varying the starting value of the background fit (indicated by the vertical line in the left panels) by ±50% in 10 steps and the background dimensionality in 3 steps by ±0.5 relative to the value shown in the left panels. The validation tool calculates for each set of parameters a distance distribution and performs a statistical analysis. The distance distribution with smallest root-mean-square deviation (rmsd) from the calculated set and therefore the best fit to the experimental data is shown in black. The grey bars represent the full variation of all calculated distance distributions, which provides an uncertainty estimation. **A)** DEER data evaluation and validation of the monomeric Bax_WT_ variants in solution presented in Figure 2b. **B)** A longer time trace (2.7 µs) increases signal fidelity with respect to the 1.7 µs time traces shown in (A) since longer distances characterized by long dipolar oscillation periods can be better resolved. However, as the validation shows, the same information content can be retrieved from both time traces of Bax_WTR1_.


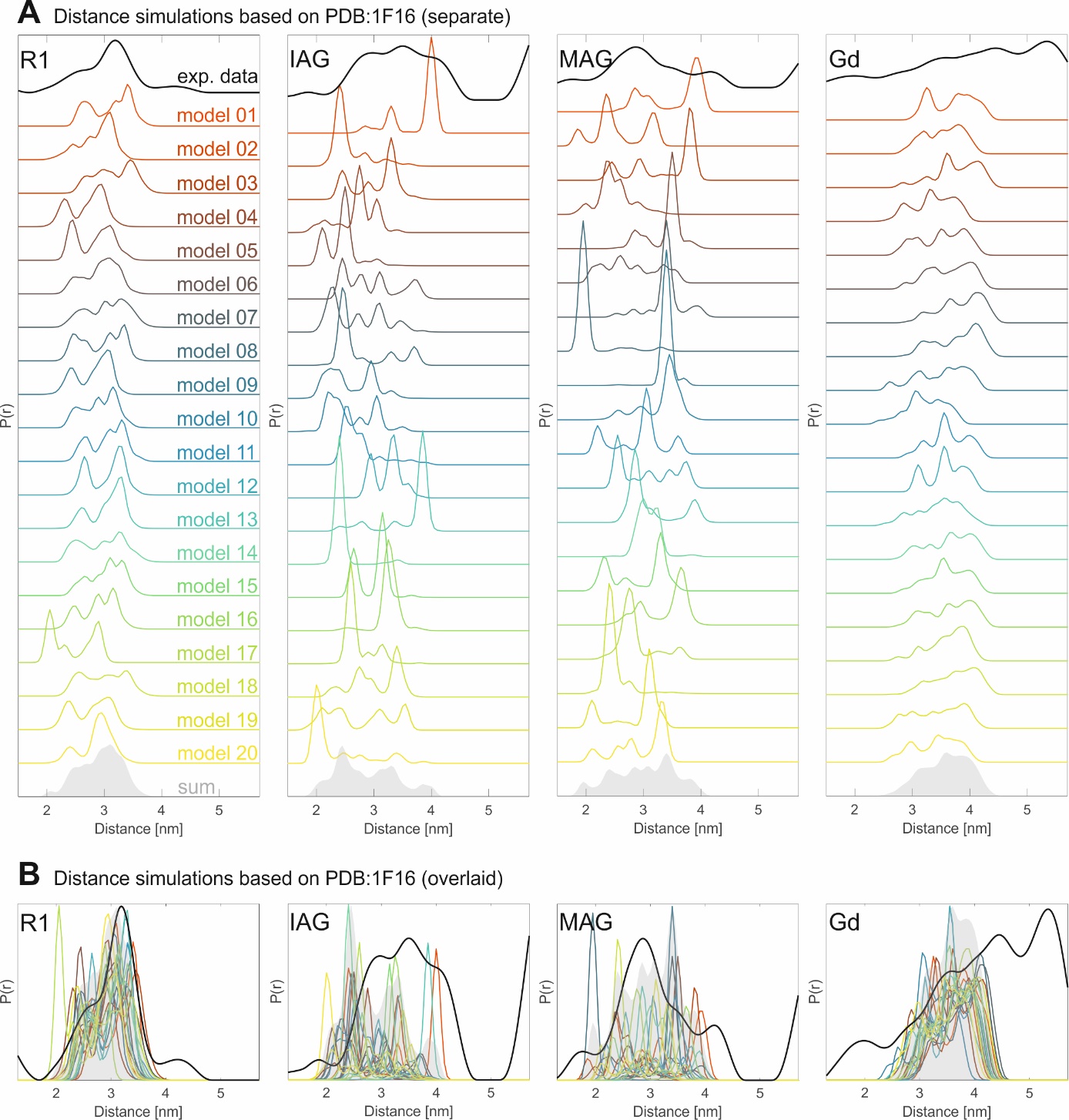


**Supplemental Figure 4 (related to Figure 2): DEER distance simulations.** MMM^5^ simulations of spin-labeled Bax_WT_ were performed using the 20 NMR structure models of monomeric inactive Bax (PDB:1F16 from^6^) in comparison with experimental data from Figure 2b (black). The grey area is the average of the distance distributions from all 20 models. The data are shown for each model separately (A) and overlaid (B). The variations in the distance distributions mainly arise from model-dependent rotamer populations of the spin labels attached at the chosen positions (C62 and C126).

**
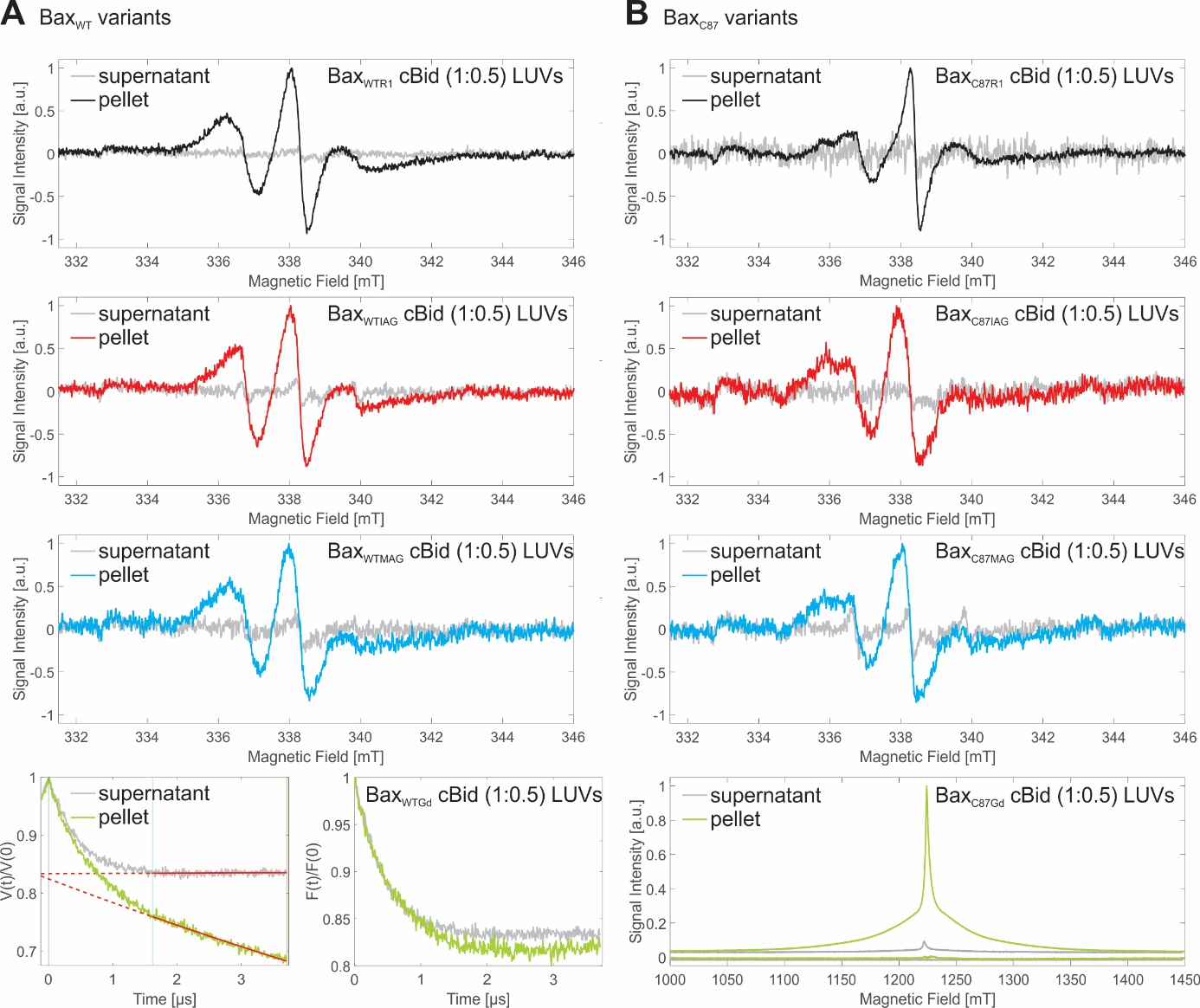
**

**Supplemental Figure 5 (related to Figure 2 and 3): Effect of spin label type on Bax membrane insertion.** After incubation of Bax with cBid (1:0.5) and liposomes (LUVs), the protein-containing liposome fraction was separated from the supernatant via ultra-centrifugation. Both pellet and supernatant were investigated via EPR. **A-B)** The six NO-containing Bax variants were investigated using cw EPR at room temperature (X band; 0.1 mT modulation amplitude; 14.5 mT sweep width). Based on the relative signal intensity, the majority of Bax_NO_ was found in the pellet fraction. **A)** Bottom panel: Bax_WTGd_ was found both in the supernatant and in the pellet. The steeper slope of the background function of the DEER time trace (red dotted lines) in the pellet indicates a higher protein concentration than in the supernatant. The similar form factors reveal that the conformation of the protein is the same in both fractions. We assume that the protein is in a physiologically not relevant conformation and membrane binding is likely facilitated by helix 9. These observations are in line with the low activity of Bax_WTGd_ seen in the calcein release assays (Figure 1f) and the unexpected distance distribution found in DEER experiments. **B)** Bottom panel: Field swept echo (FSE) spectra (Q-band; 10 K; srt = 2000 µs; 10,000 points; 450 mT sweep width; n=1; h=100; video gain 6 dB) of Bax_C87Gd_ show that the majority of the protein is found in the pellet.

**
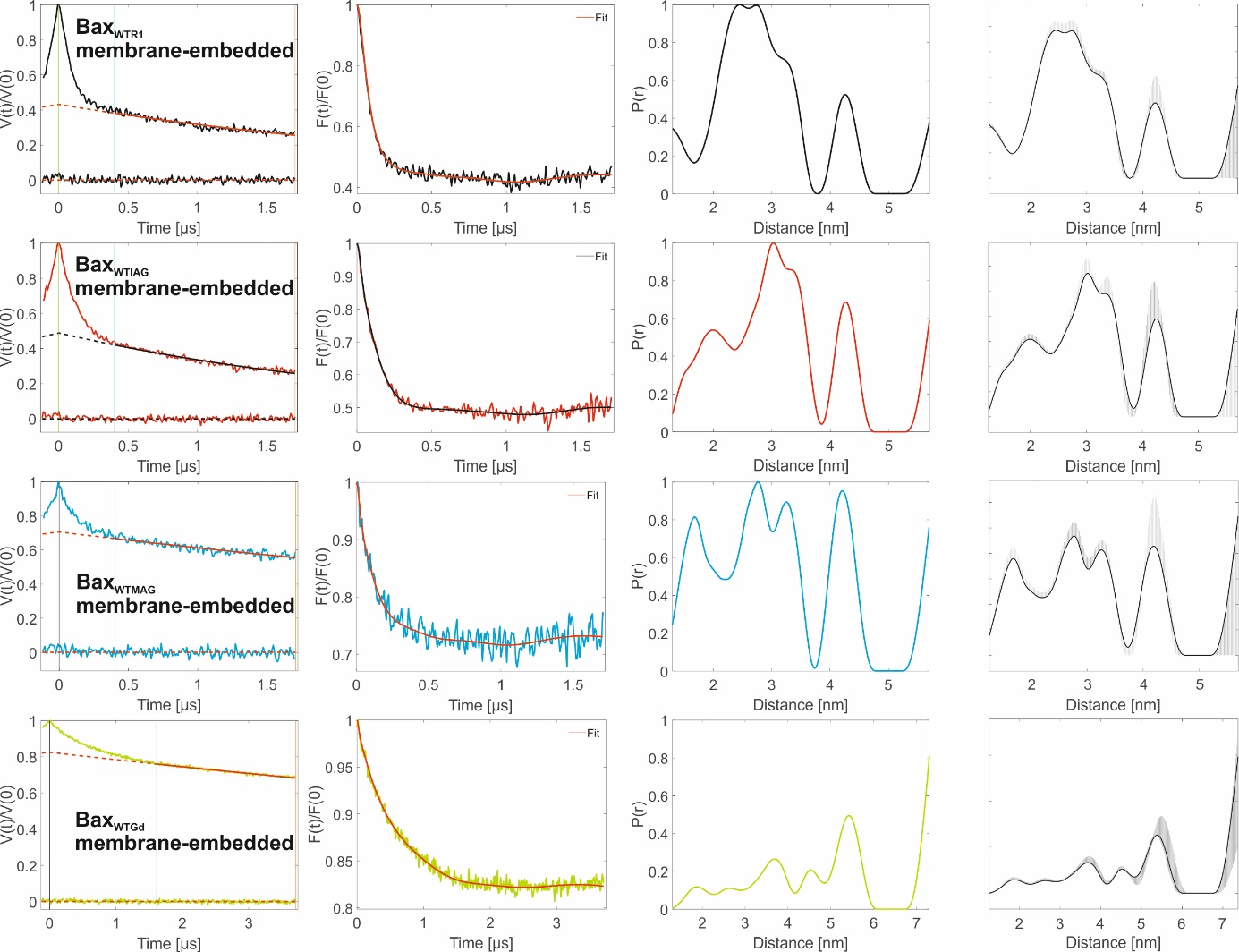
**

**Supplemental Figure 6 (related to Figure 2): DEER data evaluation and validation of the membrane-embedded Bax_WT_ variants.** DEER data evaluation and validation performed with DeerAnalysis2018^3^. From left to right: primary DEER data with background fit; form factors with fit; obtained distance distributions (Tikhonov regularization parameter 100 for NO and 1000 for Gd). Color code as in Figure 2b. Fits are shown as red or black lines; dotted regions are excluded from fitting. Right panels: Data validation performed by varying the starting value of the background fit by ±50% in 10 steps and the background dimensionality by ±0.5 in 3 steps relative to the values used in the left panels. A more detailed description of the DEER data validation is given in Supp. Figure 4.

**
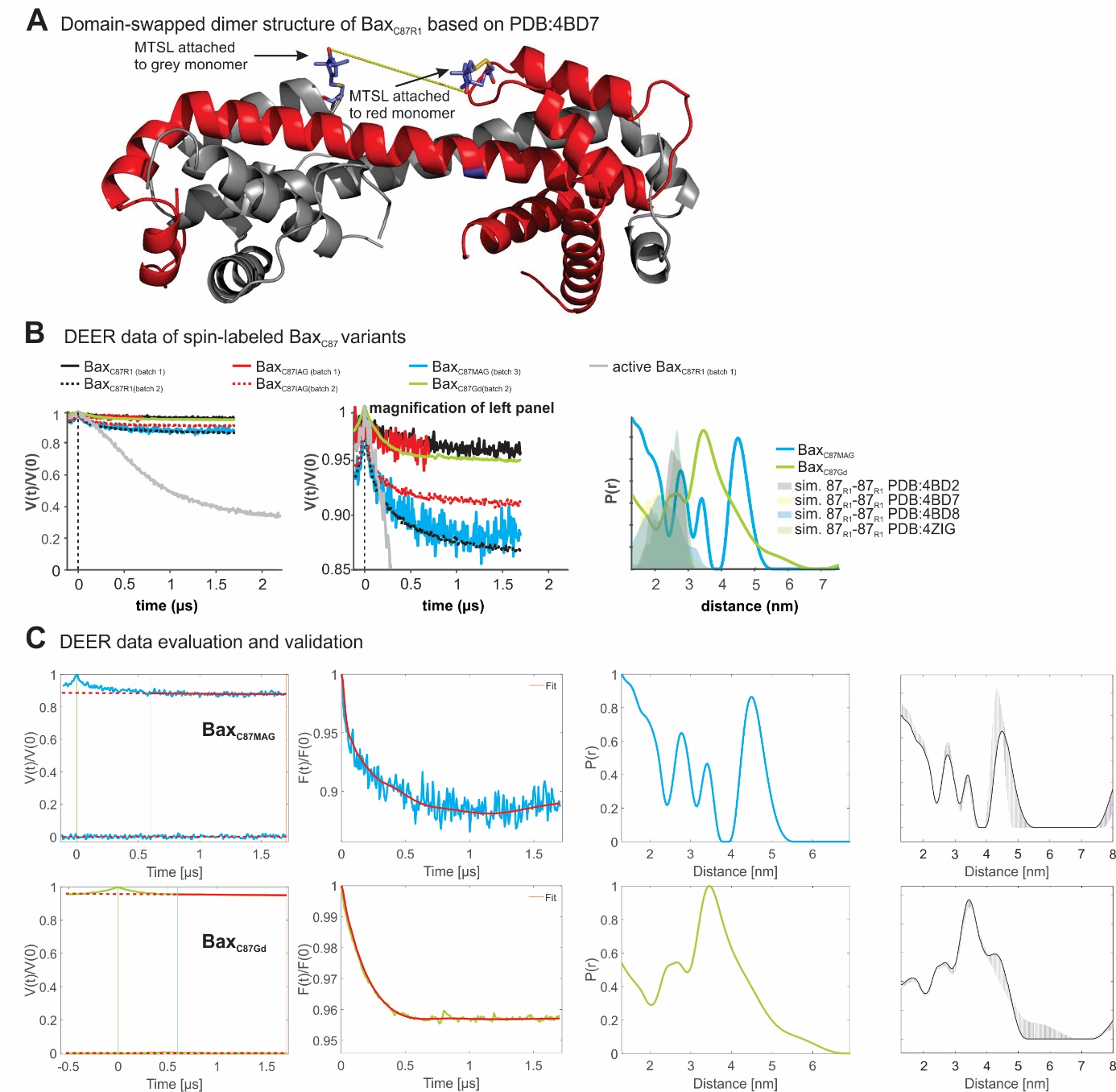
**

**Supplemental Figure 7 (related to Figure 3): Domain swapped dimer of Bax. A)** PDB structure of the domain-swapped dimer of Bax labeled with MTSL at position C87 (one monomer shown in grey, the second one in red). The structure is described as an off-pathway conformation^7^. This quaternary structure has been crystallized several times (e.g. PDB: 4BD2, 4BD7, 4BD8, 4ZIG), suggesting that it can be easily populated in vitro. **B)** Left panel: primary DEER data obtained on different batches of Bax_C87R1_, Bax_C87IAG_, Bax_C87MAG_ and Bax_C87Gd_ in solution (colors) compared to the DEER trace of active, oligomeric Bax_C87R1_ (grey). Middle panel: magnification of the left panel. Right panel: distance distributions of Bax_C87MAG_ and Bax_C87Gd_ compared to MMM simulations of the C87R1-C87R1 distance obtained with four different Bax swapped dimers structures. The small modulation depth of the primary data shows that most of the spin-labeled proteins are monomeric, but for some batches up to 10% of the population in the ensemble is engaged in dimers with distance distributions in line with the domain-swapped dimer, but clearly distinct from the active dimer. Notably, different protein batches showed different amounts of the domain-swapped dimer contaminations. **C)** DEER data evaluation and validation performed with DeerAnalysis2018^3^. From left to right: primary DEER data with background fit; form factors with fit; obtained distance distributions (Tikhonov regularization parameter 100). Color code as in (B). Fits are shown as red lines; dotted regions are excluded from fitting. Right panels: data validation performed by varying the starting value of the background fit by ±50% in 10 steps and the background dimensionality in 3 steps by ±0.5 relative to the values shown in the left panels. A more detailed description of the DEER data validation is given in Supp. Figure 4.

**
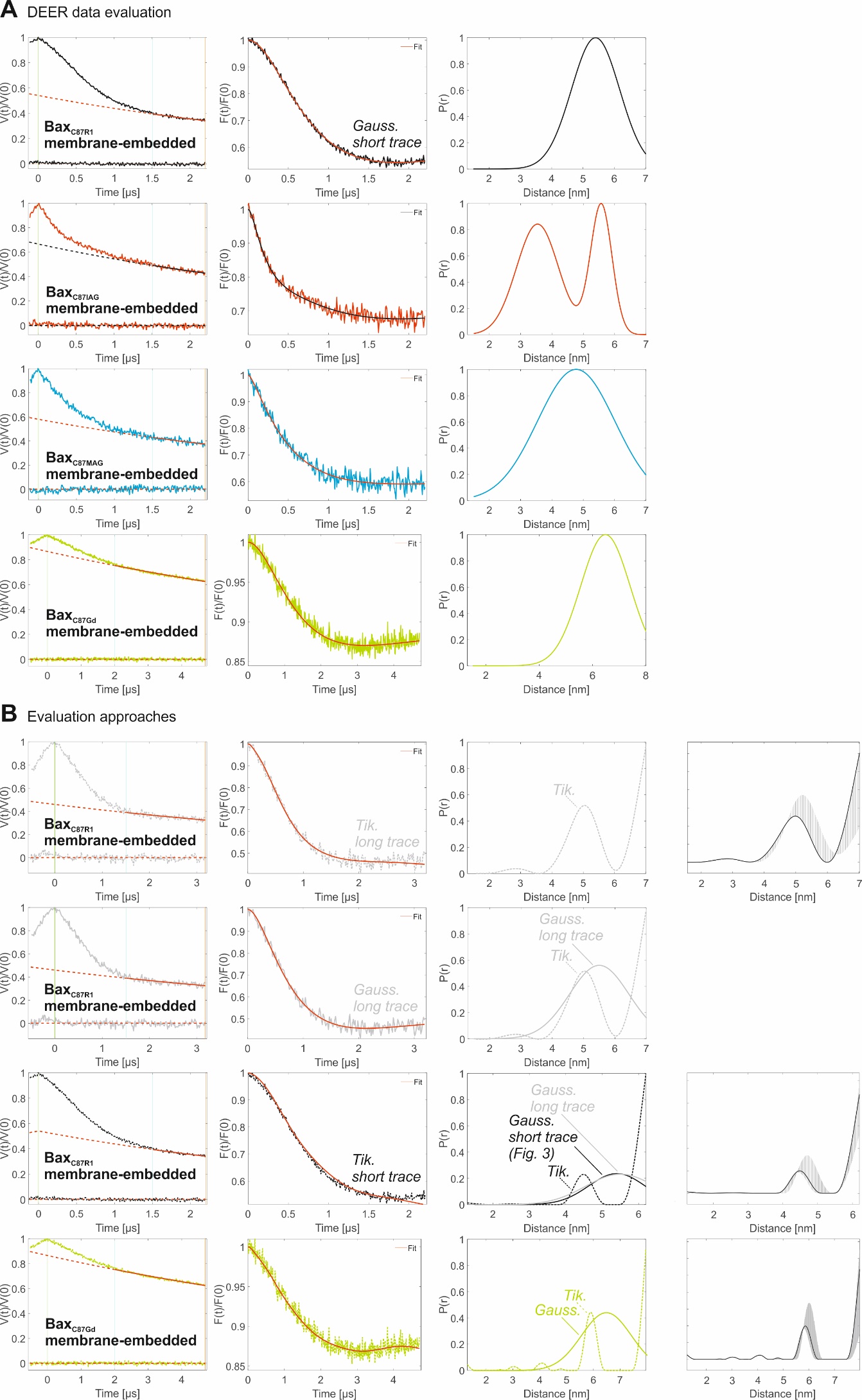
**

**Supplemental Figure 8 (related to Figure 3): DEER data evaluation of the membrane-embedded Bax_C87_ variants.** Deer data evaluation and validation performed with DeerAnalysis2018^3^. From left to right: primary DEER data with background fit; middle, form factors with fit; right, obtained distance distributions (Gaussian fit or Tikhonov regularization). Color code as in Figure 3b. Fits are shown as red or black lines; dotted regions are excluded from fitting. Right panels: validation of the distance distributions obtained from Tikhonov regularization by varying the starting value of the background fit by ±50% in 10 steps relative to the value shown in the left panels. **A)** Deer analysis of Bax_C87_ membrane-embedded variants presented in Figure 3b using one- (Bax_C87R1_, Bax_C87MAG_ and Bax_C87Gd_) or two- (Bax_C87IAG_) Gaussians. **B)** For a better comparability and to achieve a good signal-to-noise ratio, all NO time traces presented in Figure 3 were measured with the same length (2.2 µs). The sample with the lowest EPR signal determined the overall maximal length. The evaluation of long distances (> 5 nm) based on the fit of these short time traces is problematic. However, for Bax_C87R1_ a 3.2 µs time trace could be recorded (first row) and analyzed using Tikhonov regularization. A main peak at 4-6 nm is extracted which is validated by statistical analysis, and in addition, the distance probability raises towards the end of the distance distribution plot. The latter is due to the still too short trace detected, which limits the reliability of the background function. Notably, DeerAnalysis restricts the kernel functions used to fit the time-domain DEER data depending on the length of the primary traces. If the traces are too short, the distance peak extracted has not reliable mean distance and width. The one-Gaussian analysis of the same time trace (second row) is presented. The peak obtained correlates well with that obtained by Tikhonov, when considering the statistical errors. The third row shows the discrepancies in the distance distributions obtained with Gaussian (shown in panel (A) and in Figure 3) and Tikhonov fit on the short DEER trace. The Gaussian analysis of the DEER data provides a better fit of the form factor (third row vs panel (A)) and a distance distribution comparable with that obtained with the Gaussian-Tikhonov analysis of the longer time trace. Therefore, we chose to use the Gaussian analysis for the traces shown in Figure 3 (see also panel (A)). The fourth row shows the comparison between Gaussian and Tikhonov analysis on the Gd-labeled variant. In this case, due to the longer time trace detected (> 4 µs), Tikhonov (including the statistical errors) and Gaussian fits provide similar results.


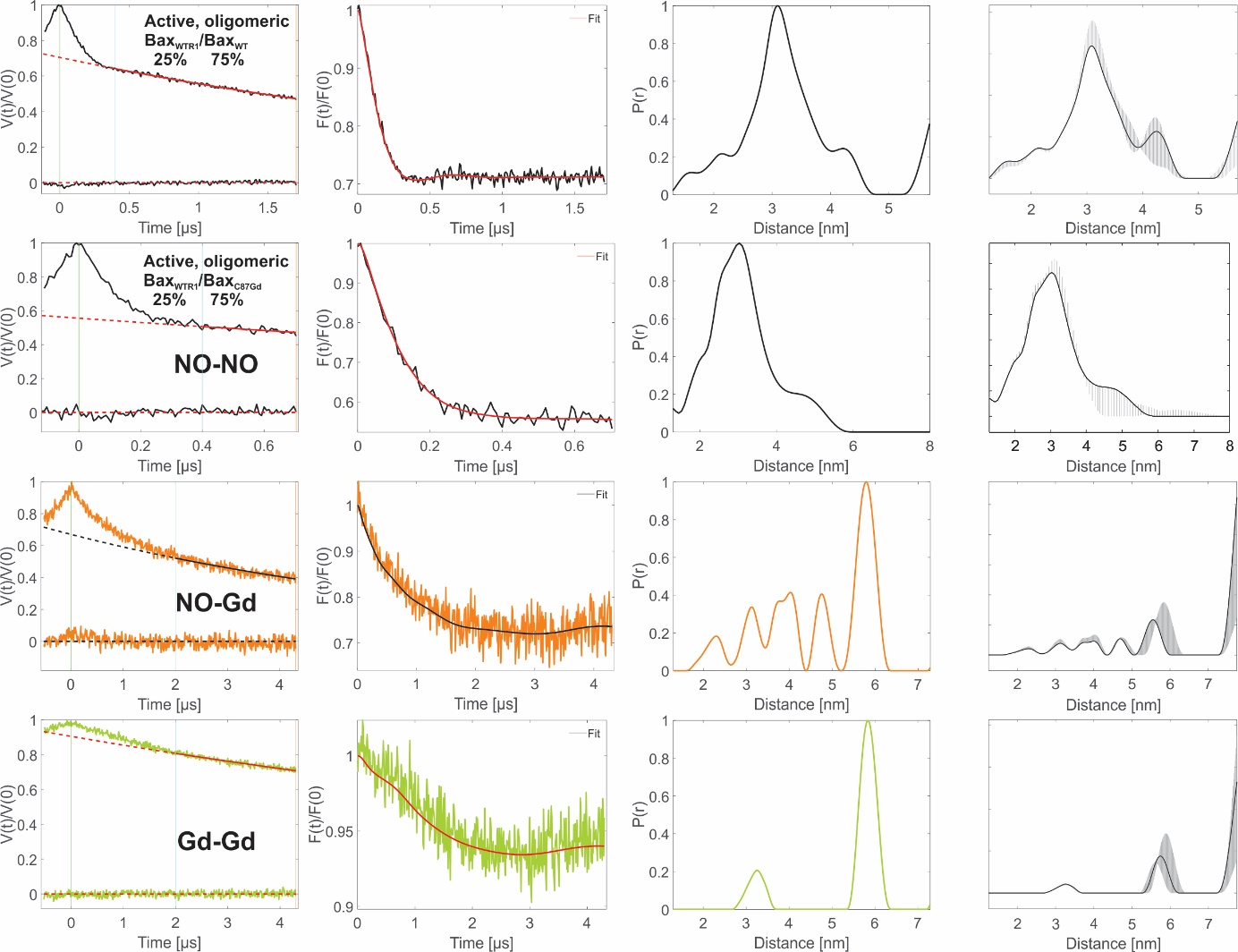


**Supplemental Figure 9 (related to Figure 4): DEER data evaluation of membrane-embedded Bax oligomers with orthogonal spin labels (MTSL and Gd).** Deer data evaluation and validation performed with DeerAnalysis2018^3^. From left to right: primary DEER data with background fit; middle, form factors with fit; right, obtained distance distributions (Tikhonov regularization parameter 100 for NO-NO and 1000 for NO-Gd/Gd-Gd). Color code as in Figure 4. Fits are shown as red or black lines; dotted regions are excluded from fitting. Right panels: data validation performed by varying the starting value of the background fit by ±50% in 10 steps relative to the values used in the left panels. A more detailed description of the DEER data validation is given in Supp. Figure 4. First row: data related to Figure 4b; second to fourth row: data related to Figure 4c.

**
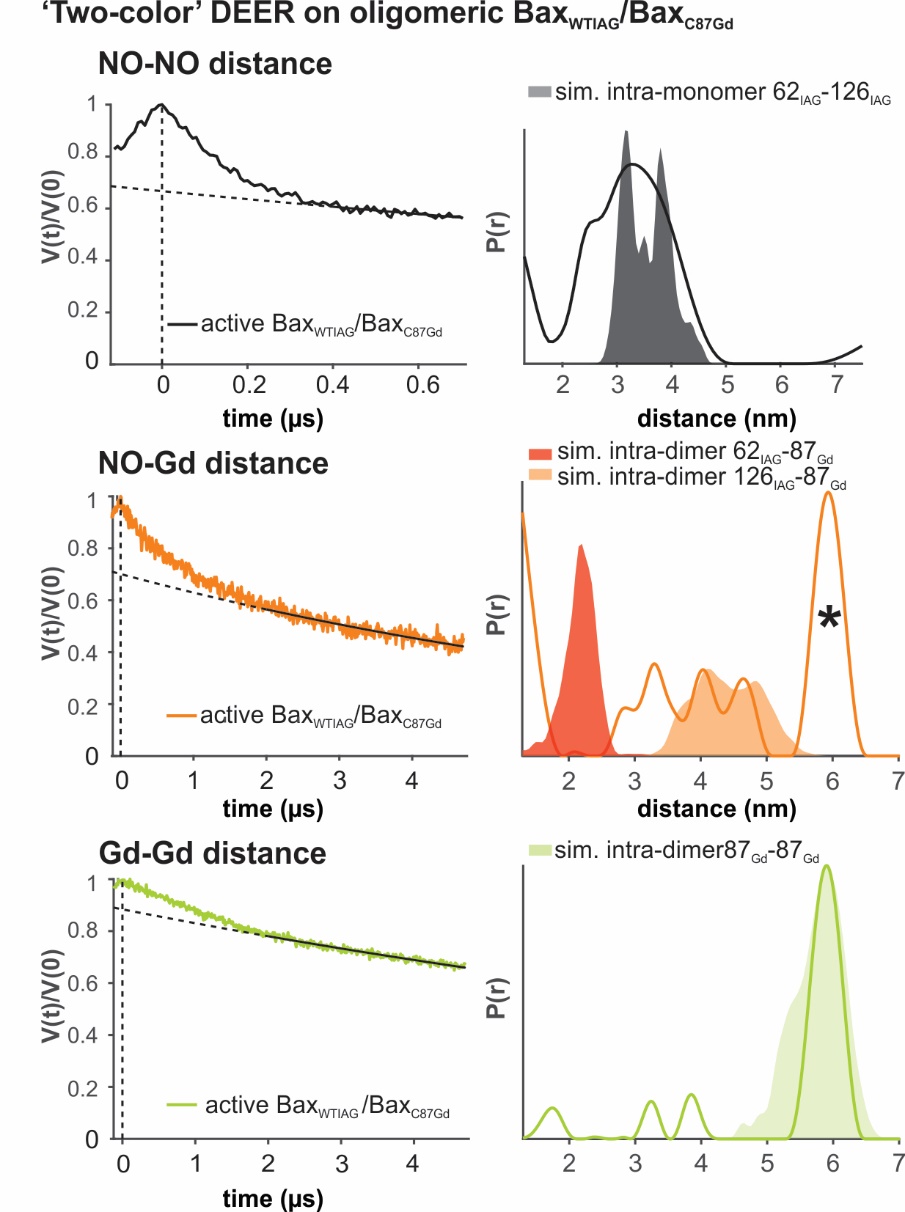
**

**Supplemental Figure 10 (related to Figure 4c): DEER data on Bax oligomers with biocompatible maleimide Gd-DOTA and IAG spin labels.** Primary data with background function (left) and distance distributions (right) obtained by Tikhonov regularization with DeerAnalysis2018^3^. The shaded areas present the corresponding MMM^5^ distance simulations based on the structures^7,8^. Upper panel, NO-NO DEER on active Bax_WTIAG_ with a 3-fold excess of Bax_C87Gd_. Central panel, NO-Gd DEER on the same sample. The asterisk highlights a possible channel cross-talk signal. Bottom panel, Gd-Gd DEER on the same sample. Validation of the distance distributions is given in Supp. Figure 11.

**
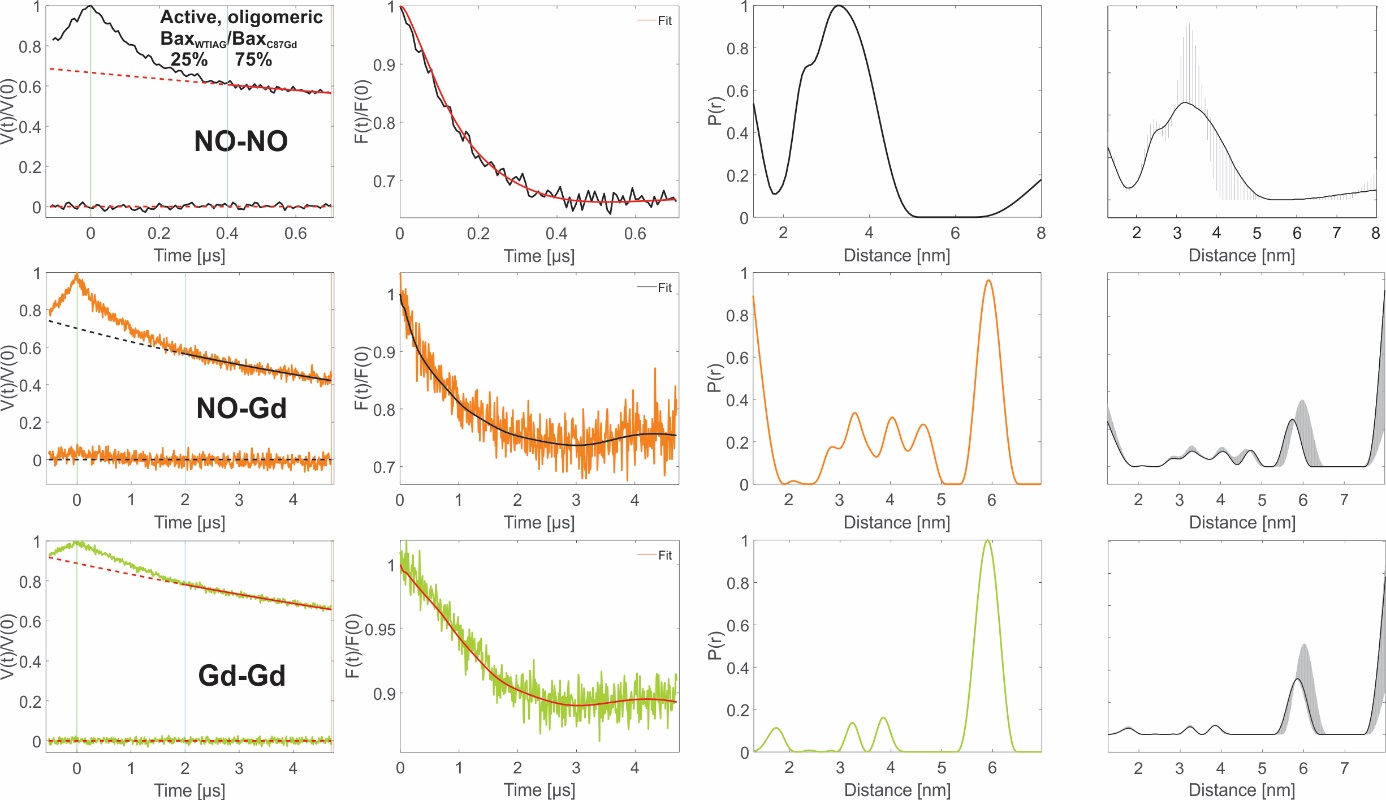
**

**Supplemental Figure 11 (related to Figure 4c and Supp. Figure 10): DEER data evaluation of membrane embedded Bax oligomers with biocompatible orthogonal spin labels (IAG and maleimide Gd-DOTA).** Deer data evaluation and validation performed with DeerAnalysis2018^3^. From left to right: primary DEER data with background fit; middle, form factors with fit; right, obtained distance distributions (Tikhonov regularization parameter 100 for NO-NO and 1000 for NO-Gd/Gd-Gd). Color code as in Supp. Figure 10. Fits are shown as red or black lines; dotted regions are excluded from fitting. Right: Data validation performed by varying the starting value of the background fit by ±50% in 10 steps relative to the values used in the left panels. A more detailed description of the DEER data validation is given in Supp. Figure 4.


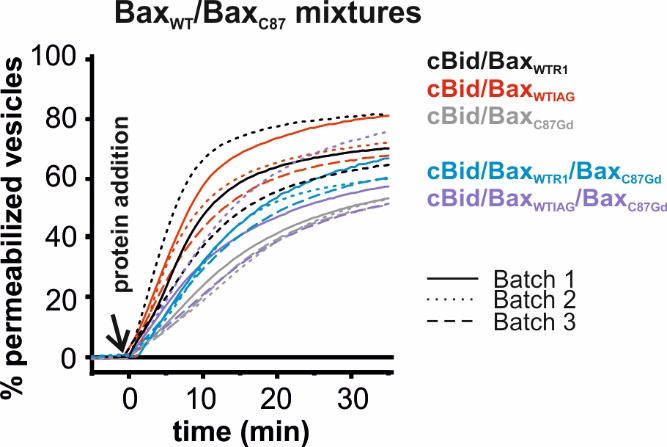


**Supplemental Figure 12 (related to Figure 4): Pore- forming assay using mixtures of Bax variants.** Experiments performed on three different batches of each Bax variant. The same batches of liposomes and cBid were used. The final Bax and cBid concentrations were 50 nM.


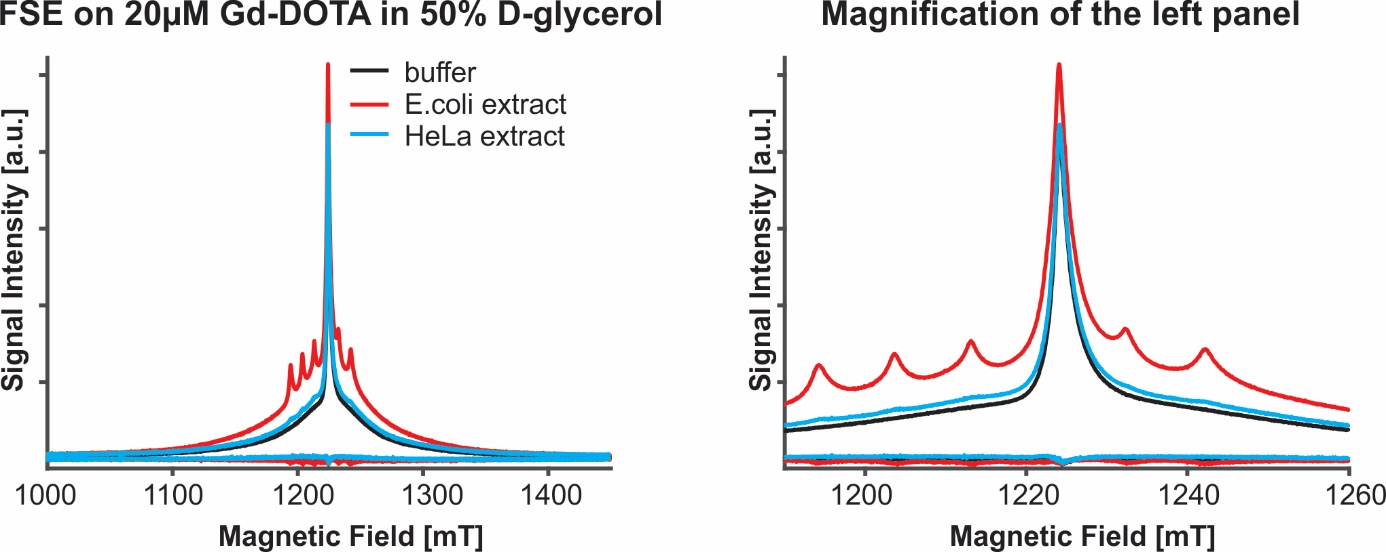


**Supplemental Figure 13 (related to Figure 5): Stability of maleimide Gd-DOTA in *E. coli* or HeLa cell extracts.** Q-band FSE spectra acquired under the following conditions: 10K; mw frequency set to the center of the resonator dip; Gaussian pulses^1^; (16-400-32) ns Hahn echo sequence; srt=2000 µs; 10,000 points; 450 mT sweep width; n=1; h=100; VG=6 dB. The spectrum detected on a sample containing 20 µM free maleimide Gd-DOTA in buffer is compared to the same amount of label incubated for 1h at RT with >6x *HeLa* cell extract or >6x *E. coli* cell extract. (Right panel shows a magnification of the central region of the FSE spectra.) Notably, *E. coli* cell extracts show additional EPR signals originating from Mn^2+^, which are negligible in HeLa cells.


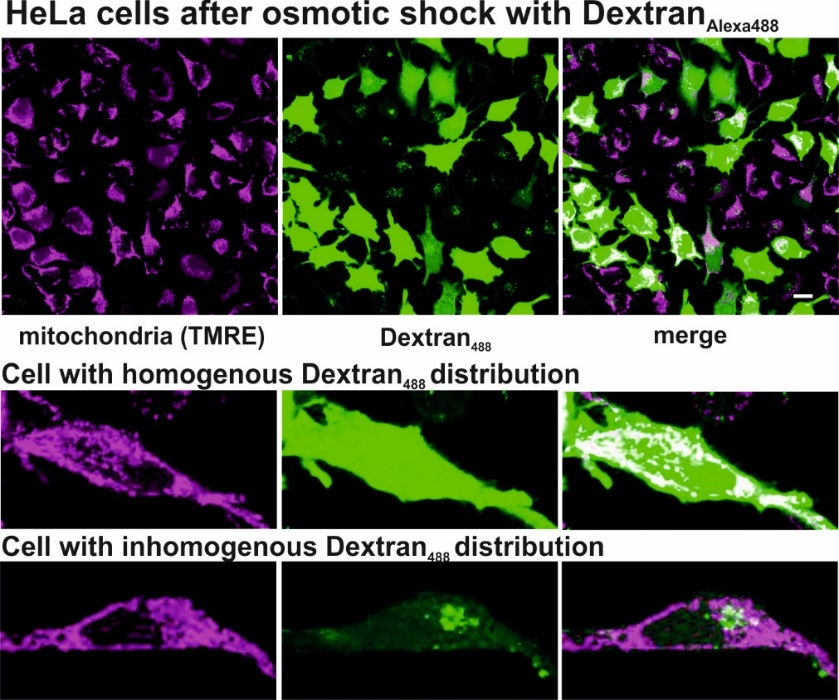


**Supplemental Figure 14 (related to Figure 6): *HeLa* cells after osmotic shock and uptake of a 10 kDa Dextran_Alexa488_.** The upper row presents overview images, the lower row enlarged sections. To visualize the mitochondria and their integrity TMRE was added as mitochondrial marker. Scalebar: 20 µm. Notably, in most cells the Dextran is cytosolic, but some cells show it enhanced in sub-cellular compartments that are not mitochondria. We surmise that these are the same compartments as the ones containing Bax_C4ATTO488_ shown in Figure 6b.


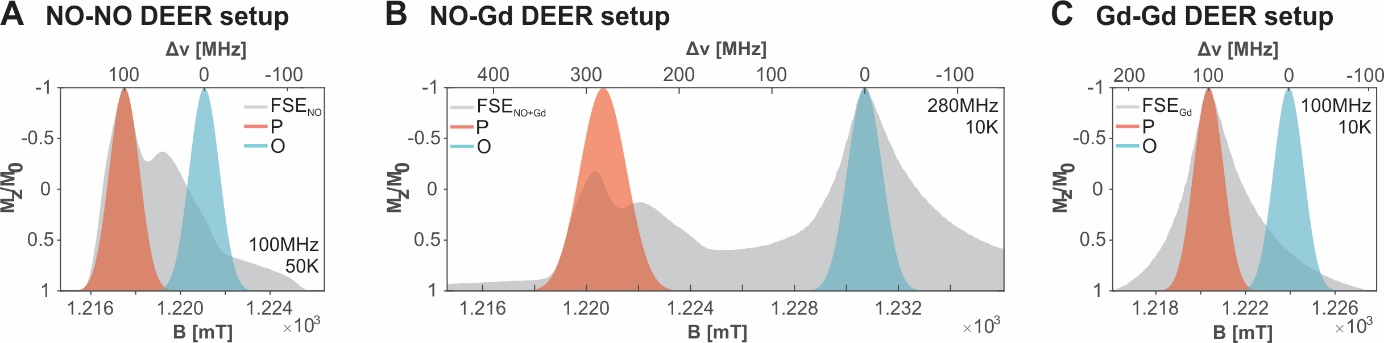


**Supplemental Figure 15: ‘Two-color-three-channel’ DEER setups.**

**A-C)** Q-band field swept echo (FSE) spectra (grey) with excitation profiles of the pump (P) and observer (O) π-pulses (simulated with EasySpin^1^) used in the specific setup. The pump pulses are depicted in red at a frequency separation Δν with respect to the observer pulses (blue). In case of orthogonal spin labeling, samples contain two spectroscopically distinguishable spin labels, in our case a nitroxide (MTSL or IAG) and a maleimide Gd-DOTA label. Orthogonality means that properties like spectral position, temperature and mw power can be used to selectively address either one of the labels.

**A)** NO-NO DEER: Δν=100MHz, all pulses 32 ns (13.6 ns FWHM) Gaussians^4^ optimized for the S=1/2 nitroxide spins, T=50 K, srt=1000 µs. The temperature acts as a filter since due to the overall faster transverse relaxation of the Gadolinium, minimal Gd signals are detectable at the spectral position of the NO. The microwave power acts also as a filter since as the spin ½ nitroxide spins require higher microwave power (12 dB) than Gd (spin 7/2) for the same length and turning angle of a pulse. Therefore, the nitroxide-optimized microwave power increases the nominal turning angle by a factor of four for the Gd spins (overflipping).

**B)** NO-Gd DEER: Δν=280MHz, 32 ns (13.6 ns FWHM) Gaussian observer pulses, 24 ns (10.2 ns FWHM) Gaussian pump pulse, performed at 10 K, srt=1000 µs. Both spin species are separately excited at very different power levels due to their spin-based distinct transition moments. However, spectral overlap could lead to simultaneous excitation of the NO and Gd with the pump pulse, creating a residual Gd-Gd dipolar frequency in the DEER trace.

**C)** Gd-Gd DEER: 10 K, srt=1000 µs, all parameters the same as in the NO-NO DEER setup. This setup avoids spectral overlap with NO.

*References*

1. Stoll, S. & Schweiger, A. EasySpin, a comprehensive software package for spectral simulation and analysis in EPR. *J. Magn. Reson.* **178**, 42-55 (2006).

2. Jeschke, G. DEER distance measurements on proteins. *Annu. Rev. Phys. Chem.* **63**, 419-46 (2012).

3. Jeschke, G. et al. DeerAnalysis2006—a comprehensive software package for analyzing pulsed ELDOR data. *Appl. Magn. Reson.* **30**, 473-498 (2006).

4. Teucher, M. & Bordignon, E. Improved signal fidelity in 4-pulse DEER with Gaussian pulses. *J. Magn. Reson.* **296**, 103-111 (2018).

5. Jeschke, G. MMM: A toolbox for integrative structure modeling. *Protein Sci.* **27**, 76-85 (2018).

6. Suzuki, M., Youle, R.J. & Tjandra, N. Structure of Bax: coregulation of dimer formation and intracellular localization. *Cell* **103**, 645-54 (2000).

7. Czabotar, Peter E. et al. Bax Crystal Structures Reveal How BH3 Domains Activate Bax and Nucleate Its Oligomerization to Induce Apoptosis. *Cell* **152**, 519-531 (2013).

8. Bleicken, S. et al. Structural model of active Bax at the membrane. *Mol. Cell* **56**, 496-505 (2014).
